# Quantitative trait loci on chromosomes 9 and 19 modulate AII amacrine cell number in the mouse retina

**DOI:** 10.3389/fnins.2023.1078168

**Published:** 2023-02-02

**Authors:** Bridget Kulesh, Rachel Bozadjian, Ryan J. Parisi, Stephanie A. Leong, Amanda G. Kautzman, Benjamin E. Reese, Patrick W. Keeley

**Affiliations:** ^1^Neuroscience Research Institute, University of California, Santa Barbara, Santa Barbara, CA, United States; ^2^Department of Molecular, Cellular, and Developmental Biology, University of California, Santa Barbara, Santa Barbara, CA, United States; ^3^Department of Psychological and Brain Sciences, University of California, Santa Barbara, Santa Barbara, CA, United States

**Keywords:** recombinant inbred strain, chromosome substitution strain, QTL mapping, SNP, INDEL, *cis*-eQTL, *Dtx4*, *Dixdc1*

## Abstract

Sequence variants modulating gene function or expression affect various heritable traits, including the number of neurons within a population. The present study employed a forward-genetic approach to identify candidate causal genes and their sequence variants controlling the number of one type of retinal neuron, the AII amacrine cell. Data from twenty-six recombinant inbred (RI) strains of mice derived from the parental C57BL/6J (B6/J) and A/J laboratory strains were used to identify genomic loci regulating cell number. Large variation in cell number is present across the RI strains, from a low of ∼57,000 cells to a high of ∼87,000 cells. Quantitative trait locus (QTL) analysis revealed three prospective controlling genomic loci, on Chromosomes (Chrs) 9, 11, and 19, each contributing additive effects that together approach the range of variation observed. Composite interval mapping validated two of these loci, and chromosome substitution strains, in which the A/J genome for Chr 9 or 19 was introgressed on a B6/J genetic background, showed increased numbers of AII amacrine cells as predicted by those two QTL effects. Analysis of the respective genomic loci identified candidate controlling genes defined by their retinal expression, their established biological functions, and by the presence of sequence variants expected to modulate gene function or expression. Two candidate genes, *Dtx4* on Chr 19, being a regulator of Notch signaling, and *Dixdc1* on Chr 9, a modulator of the WNT-β-catenin signaling pathway, were explored in further detail. Postnatal overexpression of *Dtx4* was found to reduce the frequency of amacrine cells, while *Dixdc1* knockout retinas contained an excess of AII amacrine cells. Sequence variants in each gene were identified, being the likely sources of variation in gene expression, ultimately contributing to the final number of AII amacrine cells.

## Introduction

The AII amacrine cell is a unique retinal interneuron originally appreciated for its role in transmitting scotopic rod photoreceptor signaling *via* the rod bipolar cells to retinal ganglion cells by way of ON and OFF cone bipolar cell terminals (Bloomfield and Dacheux, 2001). More recent studies have identified additional roles for AII amacrine cell circuitry in photopic signaling, for instance, in mediating cross-over inhibition between ON and OFF pathways (Demb and Singer, 2012). AII amacrine cells are narrow-field amacrine cells with a set of arboreal dendrites distributed in the ON stratum of the inner plexiform layer (IPL), where they receive glutamatergic input from multiple converging rod bipolar cell terminals, as well as forming gap junctions with ON cone bipolar cell terminals and with one another. They also display lobular terminals distributed to the OFF stratum of the IPL, where these cells form inhibitory glycinergic synapses upon the terminals of OFF cone bipolar cells and the dendrites of OFF retinal ganglion cells (Kolb and Famiglietti, 1974; Famiglietti and Kolb, 1975; Strettoi et al., 1992; Hartveit and Veruki, 2012; Marc et al., 2014). Given the restricted spread of their dendritic and lobular appendages, they are, not surprisingly then, one of the more numerous types of amacrine cell in the retina (Diamond, 2017), but just how numerous they are has received relatively little attention (Strettoi and Masland, 1996; Keeley et al., 2014; Perez de Sevilla Müller et al., 2017), as have their genetic determinants.

The present study has examined the variation in the number of AII amacrine cells across 26 RI strains derived from two inbred laboratory strains of mice, the B6/J and A/J strains, to identify the genomic control of AII amacrine cell number. Three putative large-effect QTL were identified on Chrs 9, 11, and 19, and composite interval mapping confirmed two of these, on Chrs 9 and 19, as significant based on permutation testing. Chromosome substitution strain mice in turn validated the presence of A/J strain variants increasing AII amacrine cell number on each of these two chromosomes. We identified prospective candidate genes at these two genomic loci based on several criteria, including the presence of potential coding, regulatory and splicing variants distinguishing the two genomes. We pursued two of those genes, *Dtx4* and *Dixdc1*, in further detail described below.

## Materials and methods

### Quantification of AII amacrine cell number across inbred strains

The total number of AII amacrine cells was estimated in B6/J and A/J strain mice (*n* = 7 each), in their F1 progeny B6AF1 (*n* = 9) and AB6F1 (*n* = 5), in mice of the chromosome substitution strains B6.A<9> (*n* = 3) and B6.A<19> (*n* = 3), and in 26 strains of mice from the AXB/BXA recombinant inbred strain-set (*n* = 101), all obtained from The Jackson Laboratories (Bar Harbor, ME, USA). Mice were between 4 and 8 weeks of age, and the number per individual strain is indicated in the bar histograms in the respective figures. Upon their arrival, mice were given a lethal injection of sodium pentobarbital (120 mg/kg, i.p.; Euthasol; Virbac), and once deeply anesthetized, they were intracardially perfused with 2–3 ml 0.9% saline followed by ∼50 ml of 4% paraformaldehyde in 0.1 M sodium phosphate buffer (PB; pH 7.2 at 20°C). Eyes were dissected from the orbits, and post-fixed for 15 min before being transferred to PB. Whole retinas were dissected from the eyes, taking care to ensure that the entirety of the retina was intact, for subsequent immunofluorescence labeling of the AII amacrine cells.

To identify AII amacrine cells we labeled retinas using an affinity-purified rabbit polyclonal antibody to PROX1 (dilutions, catalog numbers and RRIDs for all antibodies are listed in Table 1), followed by a donkey polyclonal antibody to rabbit IgG (H+L) conjugated to Cy3, using the following immunofluorescence protocol: (1) 3 h in a protein block of 5% normal donkey serum, (2) three rinses, 10 min each, with phosphate buffered saline (PBS), (3) incubation in primary antibodies for three nights, (4) three rinses, 10 min each, with PBS, (5) incubation in secondary antibodies for one night, and finally, (6) three rinses, 10 min each, with PBS. All steps were performed with agitation at 4°C, and all solutions were made using PBS with 1% TritonX-100.

**TABLE 1:** Antibodies used in this study.

Target	Host	Conjugate	Dilution	Cell type/Structure labeled	Catalog#	RRID
PROX1	Rabbit	None	1:1000	AII amacrine cells, Horizontal cells	PRB-238C (Covance)	RRID:AB_291595
CHAT	Goat	None	1:250	Cholinergic amacrine cells	AB144P (Millipore)	RRID:AB_2079751
VGLUT3	Goat	None	1:500	VGluT3+ amacrine cells	SC26031 (Santa Cruz Biotechnology)	RRID:AB_2187701
PKC	Mouse	None	1:250	Rod bipolar cells	05-983 (Millipore)	RRID:AB_568862
SYT2	Mouse	None	1:200	Type 2 cone bipolar cells	ZDB-ATB-081002-25 (ZIRC)	RRID:AB_10013783
TH	Sheep	None	1:1000	Dopaminergic amacrine cells	AB1542 (Millipore)	RRID:AB_90755
CALBINDIN	Rabbit	None	1:2500	Horizontal cells	PC253L (Millipore)	RRID:AB_213554
CONE ARRESTIN	Rabbit	None	1:2500	Cone photoreceptors	AB15282 (Millipore)	RRID:AB_1163387
CTBP2	Mouse	None	1:250	Ribbon synapses, ONL/INL nuclei	612044 (BD Biosciences)	RRID:AB_399431
DTX4	Rabbit	None	1:200	Unknown	AP8872a (Abgent)	RRID:AB_10560370
GFP	Rabbit	AlexaFluor488	1:1000	N/A	A21311 (Invitrogen)	RRID:AB_221477
DIG	Sheep	Alkaline Phosphatase	1:5000	N/A	11093274910 (Roche)	RRID:AB_514497
Rabbit IgG	Donkey	Cy3	1:200	N/A	711-165-152 (Jackson ImmunoResearch)	RRID:AB_2307443
Sheep IgG	Donkey	AlexaFluor488	1:200	N/A	A-11015 (Invitrogen)	RRID:AB_141362
Rabbit IgG	Donkey	AlexaFluor488	1:200	N/A	A-21206 (Invitrogen)	RRID:AB_2535792
Goat IgG	Donkey	AlexaFluor647	1:200	N/A	A-21447 (Invitrogen)	RRID:AB_2535864
Mouse IgG	Donkey	AlexaFluor555	1:200	N/A	A-31570 (Invitrogen)	RRID:AB_2536180

After immunofluorescent staining, whole retinas were mounted under a coverslip and examined on an Olympus BHS fluorescent microscope equipped with a Sony video camera and linked to a computer running Bioquant Nova Prime software (R&M Biometrics), which was used to record the position of each cell across a sample field. Four fields were sampled per retina, using a 40× objective, one in each quadrant, centered at a mid-eccentric location (0.75–1.25 mm eccentric to the optic nerve head), each sampled field being 0.032 mm^2^ in area. PROX1+ AII amacrine cells were identified in each field as having brightly labeled large nuclei residing in the innermost region of the inner nuclear layer (INL), termed the amacrine cell layer (ACL); counts from the four fields were then averaged to define a mean density of AII amacrine cells, which was then multiplied by total retinal area to generate an estimated total number. Only one retina was analyzed for AII amacrine cells in each mouse, and the same investigator was responsible for identifying AII amacrine cells in each of the four sampled fields in every retina analyzed from these 32 strains, being blind to strain identity. Strains were routinely sampled in groups of three, with individual mice of the different strains coded and then randomly intermingled (Keeley et al., 2017). The AII amacrine cell number data, for all but the chromosome substitution strain mice, have been permanently deposited on GeneNetwork^1^ under the phenotype accession identifier number 10179 (AII amacrine cell number) in the mouse AXB/BXA published phenotypes database. These data, for all but the chromosome substitution strain mice, were originally reported in an on-line supplementary appendix of another study describing the covariation between 12 different cell types across this same RI strain-set of mice (Keeley et al., 2014), and subsequently in a review focusing upon the covariation of cell number in the rod pathway (Keeley et al., 2022).

### Quantitative trait locus mapping

Because the genome of each of the RI strains contains a mix of the parental haplotypes, QTL mapping takes advantage of the nearly random recombination events over the course of inbreeding each RI strain to estimate the covariance between AII amacrine cell number and the presence of the *A* versus *B* haplotype throughout the genome. GeneNetwork implements standard methods for simple and composite interval mapping to identify QTLs, while also estimating the genome-wide *p* value of a false-positive error by randomly permuting the strain data. 2,000 random permutations were used to determine the suggestive (*p* < 0.67) and significant (*p* < 0.05) thresholds for the logarithm of the odds (LOD) score, being an index of the strength of the linkage between the variation in AII amacrine cell number and genomic locus. The options selected in the mapping module in GeneNetwork excluded the parental strains because they do not contain recombinant chromosomes and therefore cannot contribute any mapping precision, but included a weighting function to take into consideration the variability observed in the individual retinas within each RI strain. Composite interval mapping was performed in GeneNetwork to control for the variation observed at each primary QTL to determine if any secondary QTL were unveiled, using the genetic background markers at the peak of each QTL: Upk2 (Chr 9), D11Mit74 (Chr 11), and D19Mit127 (Chr 19).

### Bioinformatic analysis of candidate genes

The boundaries of the QTLs on Chrs 9 and 19 were defined using the composite interval maps. First, the peak LOD score was determined at each locus, and then the boundaries for the QTL were defined as the locations where the LOD trace was higher than the peak LOD score minus 1.5 LOD. On Chr 9, there was a recombination event in one strain that caused a large drop in the LOD score (from ∼37 to 42 Mb), creating two distinct loci. Given their proximity to one another, we analyzed both regions together as one QTL. Once each QTL was defined, a comprehensive list of genes within these regions was constructed. We then identified every sequence variant present for each of these genes, including all single nucleotide polymorphisms (SNPs), insertions/deletions (INDELs), and structural variants (SVs) discriminating the B6/J and A/J genomes, using Release-1505 (GRCm38) of the Mouse Genomes Project from the Wellcome Sanger Institute^2^ and classified their consequence using the query tool (Keane et al., 2011; Doran et al., 2016). These sequence variants were then sorted into either high-priority functional or regulatory variants as described below.

#### Functional variants

These variants were predicted to meaningfully alter protein function and included: (1) frameshift or stop codon (gained/lost) variants that would result in large truncations or disruptions of the open reading frame; (2) missense variants in which the change in amino acid resulted in a difference in side chain groups (i.e., polar amino acid in one strain and a hydrophobic amino acid in the other) and/or the altered amino acid was located in an annotated domain or membrane-spanning region; (3) small in-frame (+/-) variants that were located in an annotated domain or membrane-spanning region; and (4) splice site variants that may alter the splicing of mRNA transcripts, ultimately leading to changes in protein isoforms. Protein domain information was retrieved from UniProt.^3^

#### Regulatory variants

These variants were identified as those that may alter the overall expression of a candidate gene, without altering the protein sequence. Since potential regulatory variants were far more numerous than functional variants, and their consequences much less predictable, we attempted to prioritize them using strict criteria. These high-priority variants included SNPs and INDELs located in the proximal promoter region (defined as being within 500 bp of a transcriptional start site), in the 5′ untranslated region (UTR) or in the 3′ UTR of a gene, as well as being found within evolutionarily conserved regions (ECRs); additionally, we prioritized SVs at any location within a gene that were also found in ECRs. We defined such regions using the default settings of the Evolutionary Conservation of Genomes Browser^4^ to determine if a 100 b region surrounding the variant of interest was conserved across two or more species. Furthermore, for variants found in the 3′UTR, we consulted a microRNA resource to determine if they disrupted or created a predicted microRNA binding site (Betel et al., 2008).

#### Ranking candidates

Information regarding the expression patterns and known functions of genes at each locus was collected using a variety of databases. Functional information was found on NCBI Gene^5^ and DAVID Bioinformatics^6^. Various expression datasets were used to determine developmental and adult gene expression, including the GeneNetwork adult whole eye and retina microarray gene expression databases (GN Accession #GN210 and #GN302, respectively); the SAGE database of developmental and adult retinal gene expression^7^ (Blackshaw et al., 2004); gene expression data of AII amacrine cells at P7 (Kay et al., 2012); and single cell gene expression data for adult AII amacrine cells (Macosko et al., 2015). Genes that (a) had been shown to be expressed during retinal development and/or in AII amacrine cells, and (b) to modulate biological processes likely to affect cell number, and (c) that contained multiple high-priority variants were regarded as the highest priority for further consideration. The results of these analyses are summarized in Supplementary Tables 1, 2, where a “YES” in any category indicates at least one piece of affirming evidence was identified, while a “NO” indicates either the evidence found did not meet our criteria, or that no bioinformatical data was found.

### Analysis of *Dtx4*

#### *In situ* hybridization

RNA *in situ* hybridization was performed to examine the spatial expression of *Dtx4* in mature retina following an established protocol (Palop et al., 2011; Kautzman et al., 2018), and using riboprobes specific to *Dtx4* that were generated using PCR and *in vitro* transcription (Ghafoory et al., 2012). Primers were designed to amplify a 232 bp sequence of *Dtx4* mRNA and were coupled to adaptor sequences containing the T3 (F primer) and T7 (R primer) RNA polymerase promoter sites (Table 2). PCR products were amplified using cDNA template generated from mature B6/J retinal mRNA, and the resulting PCR product was subsequently purified and used for *in vitro* transcription reactions using digoxigenin (DIG) labeled nucleotides (#11277073910; Roche). Antisense (experimental) and sense (control) riboprobes were generated using T7 or T3 RNA polymerase (#P2075 and #P2083; Promega), respectively.

**TABLE 2:** Primers used for the development of molecular tools in this study.

Purpose	Direction	Adaptor (5′-3′)	Gene-specific sequence (5′-3′)
*Dtx4 in situ* probe	Forward	GAAATAATTAACCCTCACTAAAGGG	TGCTGATTGCCTCCGGGGTT
	Reverse	GAAATTAATACGACTCACTATAGGG	TTGGTGGTCTTGCGGCTCGT
*Dtx4* expression plasmid	Forward	TAAGCAGAATTC	ATGCTCCTGGCCTCGGCAG
	Reverse	TGCTTAGCGGCCGC	TCAGTCCTTTTCATGGGAAGTGCTG
*Dixdc1* full-length expression plasmid	Forward	CCGCTCGAGC	GCCTTTGTGTACAGAGGGAA
	Reverse	AAGGAAAAAAGCGGCCG	CTCAGTGGGCTAAGGACAAGC
*Dixdc1* truncated expression plasmid	Forward	None	GTTTTCCATGATGACGATGC
	Reverse	None	CAACAATCTCGATGAGATAG
*Dixdc1* missense expression plasmid	Forward	None	CTGAAGGAATGGAGAACAGAAC
	Reverse	None	AGGGAATGATCACGAAATC

Mice at approximately 2 months of age were euthanized *via* an i.p. injection of Euthasol and eyes were then dissected and lightly fixed by immersion in 4% paraformaldehyde in PB for 30 min while maintaining an RNase-free environment. Retinas were dissected and embedded for sectioning at 20 μm on a Microm HM560 cryostat (Thermo Fisher Scientific) and serial sections were mounted on glass slides. Sections were digested and acetylated, incubated overnight at 65°C with the purified *Dtx4* riboprobes (500 ng probe/mL) to hybridize with endogenous *Dtx4* mRNA, and then labeled overnight at 4°C with sheep antibodies to digoxigenin conjugated with alkaline phosphatase to detect the probes. Alkaline phosphatase activity was assessed by developing the sections in a solution of NBT/BCIP (#11681451001; Roche) until a purple/blue precipitate was visible, then subsequently dehydrated in ethanol, cleared with Neo-Clear (#109843; Millipore), and mounted under a coverslip. An Olympus BHS microscope equipped with a video camera and a 20× objective was used to image serial sections labeled with antisense and sense riboprobes while using identical illumination and camera settings, and the resulting micrographs were contrast-enhanced in Photoshop using identical parameters.

#### *In vivo* electroporation

The pCAGIG expression plasmid (RRID:Addgene_11159) was modified to overexpress *Dtx4* as well as *gfp* in early postnatal retinas, using an electroporation protocol originally developed by Matsuda and Cepko (2004, 2007). The *Dtx4*-encoding plasmid was created by amplifying the coding sequence of *Dtx4* (NM_172442.3) from a pYX-Asc vector containing *Dtx4* cDNA (Clone ID: 6405881; GE Healthcare Dharmacon) using gene-specific primers, which included adapters for the EcoRI (F primer) and NotI (R primer) restriction enzyme sites, and then cloning the resulting fragments into the pCAGIG backbone (Table 2). A small volume (0.7 μL) of either *Dtx4*-encoding or control plasmids (at a concentration of 1.5 μg/μL) was injected into the subretinal space of CD1 (RRID:IMSR_CRL:022) mouse pups on the day after birth (injecting only one eye from each animal), and then transfected into dividing cells *via* electroporation using an Electro Square Porator (Model 830; BTX), following the procedure previously described by us (Kautzman et al., 2018). Pups were then returned to their dam and left to develop until 3 weeks of age, at which point an i.p. injection of Euthasol was administered, and the electroporated eye from each pup was removed and immersed in 4% paraformaldehyde in PB for 30 min.

Fixed retinas from electroporated mice were embedded in 5% agarose in PB and then sectioned radially on an EasySlicer (Pelco) at a thickness of 200 μm. Sections were immunolabeled using a rabbit polyclonal antibody to GFP conjugated to AlexaFluor 488 (following the protocol described above) to amplify the GFP signal which indicated the presence of the pCAGIG plasmid; sections were additionally labeled with Hoechst 33342 (1:1000; #H3570; Invitrogen) to identify the nuclear layers of the retina, as well as assess the retinal architecture. For each retina, a single micrograph was taken from every section that contained GFP+ cells and displayed a normal retinal architecture (i.e., removed from the site of the injection) using an Olympus Fluoview1000 laser scanning confocal microscope. Micrographs from both conditions were coded and then randomly intermingled, to conceal identity and to prevent batch effects. Each GFP+ cell was then classified as residing in the outer nuclear layer (ONL), the outer half of the INL, or the inner half of the INL, the latter being the operational definition of the ACL for this analysis. GFP+ cells were rarely observed in the ganglion cell layer (GCL), and were excluded from the quantification. For each retina, the numbers of positive cells in the ONL and INL were summed across the sections, and the total number of cells in the ACL was expressed as a percentage of all GFP+ cells.

### Analysis of *Dixdc1*

*Dixdc1*-knockout (KO) mice, carrying the B6.129-*Dixdc1^TM1Bnrc^*/J allele (Kivimae et al., 2011), were obtained from The Jackson Laboratory (#029504; RRID:IMSR_JAX:029504) and bred as heterozygotes at UCSB to obtain KO and littermate wildtype (WT) mice. At 6–8 weeks of age, mice were perfused, eyes were harvested, and retinas were immunolabeled as described above. The numbers of AII amacrine cells and six additional neuronal cell types were determined from whole retinas (cholinergic amacrine cells, VGluT3 amacrine cells, dopaminergic amacrine cells, rod bipolar cells, Type 2 cone bipolar cells and horizontal cells), while changes to the retinal architecture were examined from 200 μm thick retinal sections; all tissue was labeled using the immunofluorescence protocol described above. Primary and secondary antibodies used to label *Dixdc1* KO and WT retinal tissue are listed in Table 1. In addition, either NeuroTrace 530 (1:250, #N21482; Invitrogen) was used to label neuronal cell bodies or Hoechst 33342 was used to label nuclei; when used, these were added to the cocktail of primary antibodies.

#### Cell counts

For all cell types except for the dopaminergic amacrine cells, immunolabeled retinal wholemounts were mounted under a coverslip and imaged using an Olympus Fluoview1000 laser scanning confocal microscope. Eight fields were sampled from each retina (at one central and one peripheral location per quadrant) using a 40× objective, and all eight image stacks per retina from all the KO and WT mice were randomly interleaved and counted by an individual, who was therefore blind to the identity of each sampled field. An average density was calculated for each retina and multiplied by retinal area to estimate total cell number. AII amacrine cell nuclei were identified using antibodies to PROX1 (as above) in a sample field of 0.057 mm^2^; horizontal cell nuclei were identified as large PROX1+ nuclei in the outermost region of the INL in a sample field of 0.101 mm^2^; cholinergic amacrine cells were identified as CHAT+ cells in both the INL and GCL in a sample field of 0.176 mm^2^; VGLUT3+ amacrine cells were identified in the INL in a sample field of 0.045 mm^2^; rod bipolar cells were identified from PKC+ axon stalks coursing through the IPL in a sample field of 0.011 mm^2^; and Type 2 cone bipolar cells were identified by their SYT2+ axonal stalks coursing through the IPL in a sample field of 0.045 mm^2^. The entire population of dopaminergic amacrine cells within each retina was quantified from retinal whole mounts using the Olympus fluorescence microscope and systematically scanning across the entire retina to identify every TH+ cell using BioQuant software (Keeley et al., 2017); each retina was randomally assigned a code before quantification to blind the counter to condition.

#### Quantitative (q)PCR

Fresh retinas were dissected from the eyes of anesthetized P1, P5, P10, and adult B6/J and A/J mice and placed into RNAlater (BioRad), taking care to avoid RNase contamination. Adult samples were comprised of both retinas from individual animals, while developmental samples were comprised of pooled retinas from mice in the same litter (∼3–4 mice). RNA was purified from each sample using an RNeasy Mini Kit (#74104; Qiagen), and single stranded cDNA was produced from these samples using an iScript cDNA synthesis kit with oligo (dT) and random primers (#1708890; BioRad). Sso Advanced Universal SYBR Green Supermix was used to determine the relative expression of full-length *Dixdc1* transcripts (using primers directed to the common 3′UTR) to the amount of the truncated transcripts *via* qPCR. Adult and developmental samples were assessed separately. For each gene, every sample was run on a single plate in triplicate using the CFX96 qPCR thermal cycler (BioRad). Glyceraldehyde 3-phosphate dehydrogenase (*Gapdh*) and β-2 microglobulin (*β2m*) were used as internal control genes for normalizing *Dixdc1* expression to compare quantities across samples (as described in Keeley et al., 2012). Details for the qPCR primers are listed in Table 3.

**TABLE 3:** Primers used for quantitative PCR.

Purpose	Direction	Primers (5′-3′)	Product length	Annealing temp
*Gapdh*	Forward	AATGTGTCCGTCGTGGATCTGA	117 bp	63°C
	Reverse	AGTGTAGCCCAAGATGCCCTTC		
*β2m*	Forward	GGAGAATGGGAAGCCGAACATAC	143 bp	63°C
	Reverse	AGAAAGACCAGTCCTTGCTGAAG		
*Dixdc1* 3′UTR (full length)	Forward	TGCATCCACTTCCAGGTTCC	86 bp	61.5°C
	Reverse	AACCGAAGGCCTTAACCACC		
*Dixdc1* third intron (truncated)	Forward	ACAGACCTCACTCTCCAGTCT	161 bp	63°C
	Reverse	AACAGCGTCGGGAAATACCTA		

#### Luciferase assay

Three expression plasmids were created to test the effect of DIXDC1 protein variants on β-catenin signaling *in vitro*. Initially, the entire *Dixdc1* coding sequence (full-length) was amplified from cDNA generated from mRNA collected from the brain of an adult B6/J mouse, using gene-specific primers including adapters for the XhoI (F primer) and NotI (R primer) restriction enzyme sites (Table 2). Purified amplicons were cloned into the multiple cloning site of the pTargeT Mammalian Expression Vector (#A1410; Promega), which uses a CMV enhancer/promoter to drive expression of the coding sequence and contains a SV40 late poly(A) tail to improve transcript stability. Sanger sequencing revealed that the cloned transcript of *Dixdc1* matched the RefSeq annotated transcript NM_001374656. To create a truncated *Dixdc1*-encoding plasmid, the region of the coding sequence after the second exon of this transcript was removed from this full-length *Dixdc1*-encoding plasmid using a Q5 Site-Directed Mutagenesis Kit (#E0554S; NEB) with primers designed using the NEBaseChanger tool^8^ ; the resulting truncated protein product shares the first 63 amino acids of full-length DIXDC1 (RefSeq annotated protein NP_835219), while lacking the subsequent 622 amino acids of the C-terminal, which include a part of the calponin homology domain, the actin binding domain, the coiled-coil domains, and the DIX domain. To create a *Dixdc1*-encoding plasmid that contains the A/J missense variant, the SNP was introduced in order to change amino acid 252 from an isoleucine to a threonine in the full-length encoding plasmid again using a Q5 Site-Directed Mutagenesis Kit. In addition to the DIXDC1 expression plasmids, a plasmid that expresses Disheveled 2 (DVL2) under a CMV promoter (#123587; Addgene; RRID:Addgene_123587) was also used to stimulate WNT signaling in transfected cells (Choi et al., 2019).

For each luciferase experiment, HEK293T cells were seeded in 24-well plates coated with gelatin at a density of 2 × 10^6^ cells/well. Cells were grown in DMEM:F12 medium (#11320033; Thermo Fisher Scientific) with 20% Newborn Calf Serum (#160110167; Thermo Fisher Scientific) and penicillin/streptomycin (100 U/mL and 100 ug/mL, respectively; #15140122; Thermo Fisher Scientific) for 2 days, after which cells were transfected with a combination of plasmids using TurboFect (#R0531; Thermo Fisher Scientific). Each well received a combination of four types of plasmids: (1) a DIXDC1 expression plasmid (control, full-length, truncated, or missense), (2) a DVL2 expression plasmid (control or protein expressing), (3) the β-catenin reporter firefly luciferase-expressing plasmid (Super 8x TOPFlash; RRID:Addgene_12456), and (4) the transfection control *Renilla* luciferase-expression plasmid (pRL-null; #E2271; Promega). Except for the latter, which was transfected at 20 ng per well, all plasmids were transfected at molar equivalents such that the total mass of transfected plasmids did not exceed 1,000 ng per well. Two days post-transfection, protein lysates were collected from each well using Glo Lysis Buffer (#E2661; Promega) and transferred in equal volumes (45 μL) to four wells of a 96-well plate; two wells were used to assess firefly luciferase luminescence using Steady-Glo reagent (#E2510; Promega), and two wells were used to assess *Renilla* luciferase luminescence using *Renilla*-Glo reagent (#E2710; Promega). Luminosity readings were taken using a Tecan Spark plate reader, and were normalized as follows: technical repeats were averaged, and then firefly luciferase values were normalized by *Renilla* luciferase values per well. For each of the four DIXDC1 conditions, the wells receiving DVL2-encoding plasmids were normalized by the average value for the wells that did not receive the plasmid, to determine the increase over baseline β-catenin signaling caused by activating the WNT pathway. This normalization was performed for each of six plates, as each plate contained HEK293T from a different passage. Each well was treated as an independent biological sample, resulting in 18 total wells per condition; one well from the missense DIXDC1 condition was excluded as it was an extreme outlier, resulting from abnormally low *Renilla* luminosity values.

### Statistics

A one-way ANOVA was used to examine for differences in AII amacrine cell number between mice of the following strains: parental A/J and B6/J strains, their reciprocal B6AF1 and AB6F1 offspring, and consomic B6.A<9> and B6.A<19> strains. Comparisons using *post-hoc* Tukey tests assessed whether the parental B6/J and A/J strains and their reciprocal F1 strains each differed from one another, and whether either of the two consomic strains differed from the parental B6/J strain. Because these results are considered at different locations in the Results section, the B6/J strain data are reproduced in the respective histograms, but the statistical analysis is based on a single ANOVA and associated Tukey tests. One-way ANOVA and *post-hoc* Tukey tests were also used to examine differences in AII amacrine cell number between all of the different RI strains, and between the different groups of RI strains sorted by haplotype at Chrs 9 and 19.

Student’s two-tailed *t*-tests were used to assess statistical significance for all comparisons between *Dixdc1* KO and littermate WT mice, and between mice given *Dtx4*-encoding and control plasmids. The levels of *Dixdc1* transcript expression determined from qPCR experiments were assessed for statistical significance between the B6/J and A/J strains of mice across the three developmental ages, and for any interaction between strain and age, using two-way ANOVAs followed by *post-hoc* Tukey tests. Adult parental strain *Dixdc1* expression levels, run separately, were compared using Student’s two-tailed *t*-tests. Differences in β-catenin signaling across conditions in the *in vitro* luciferase assay were assessed using a one-way ANOVA followed by planned comparisons using *post-hoc* Tukey tests. An alpha threshold of 0.05 was used for determining statistical significance in all cases, indicated by an asterisk. Individual *p* values are indicated in the text.

## Results

### AII amacrine cell number varies across the 26 recombinant inbred strains

**FIGURE 1:**
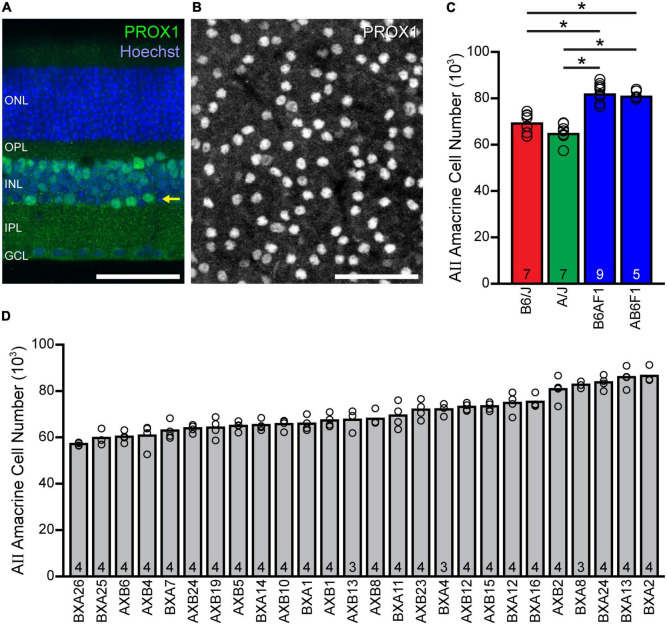
AII amacrine cell number shows considerable variation across mouse strains. **(A)** Cross-section showing the stratification of AII somata (arrow), immunolabeled using PROX1 antibodies, at the inner nuclear layer (INL)/inner plexiform layer (IPL) boundary. **(B)** Wholemount preparation showing the somal distribution of AII cells labeled for PROX1 within this stratum. Calibration bars = 50 μm. **(C)** Counts of PROX1-labeled AII cells in wholemount preparations reveal slight differences in total cell number between the parental B6/J and A/J strains of mice, as well as between their two reciprocal F1 offspring (B6AF1 and AB6F1), yet there exists a large difference between each parental strain and each F1 strain. **(D)** The total number of AII amacrine cells varies across the 26 recombinant inbred strains of the AXB/BXA strain-set, by nearly 30,000 cells. Note that the within-strain variation is meager when compared to the across-strain variation, and that the latter variation is gradual, indicative of a polygenic trait. n = the number of retinas sampled (from the supplementary appendix in Keeley et al., 2014).

AII amacrine cells in the mouse retina, which can be reliably labeled with antibodies to PROX1 (Perez de Sevilla Müller et al., 2017; Keeley and Reese, 2018), are positioned along the inner margin of the INL (Figure 1A, arrow), extending their processes across the entire depth of the IPL. The planar distribution of their somata lends itself to imaging the population in wholemount preparations, where the labeling is robust in all AII cells (Figure 1B). Estimates of the total number of AII amacrine cells show an average total of 69,223 cells in the B6/J retina and 64,777 cells in the A/J retina (Figure 1C), a non-significant difference (*p* = 0.241) of about 4,500 cells representing about 7% of the number present in each strain. Curiously, while the two F1 generation strains, B6AF1 and AB6F1, produced by crossing the parental inbred strains have very similar average numbers, being 82,050 and 80,882 cells (*p* = 0.992), respectively, each has a significantly larger population of AII amacrine cells (∼21% more) than either of the parental strains (*p* < 0.001). These results indicate the presence of countervailing genetic determinants in each parental strain that are unmasked in the heterozygous condition.

Given such large variation between the F1 strains versus the parental strains, we examined AII amacrine cell number in the AXB/BXA recombinant inbred strain-set derived from these same parental strains, with the expectation that variation across the RI strains would map to discrete genomic loci containing the source(s) of this variation. Indeed, these RI strains showed an even larger variation in average total number (Figure 1D), from a low of 57,141 cells, in the BXA26 strain, to a high of 86,557 cells, in the BXA2 strain, a difference of ∼29,400 cells, or a 51% increase from the lowest to the highest strain. A one-way ANOVA and *post-hoc* Tukey tests confirmed statistically significant differences amongst the various strain comparisons (Supplementary Figure 1). Note that the variation within each strain was meager (Figure 1D), the coefficient of variation averaging 0.05 across all strains, relative to the conspicuous variation across the strains. As previously reported, the heritability (*h*^2^) of this trait was calculated to be 0.74, indicating nearly three-quarters of the variation in AII amacrine cell number across these 101 RI mice can be ascribed to an effect of genotype (Keeley et al., 2014). Note as well that the variation across the strains is gradual (Figure 1D), although there are a couple of pronounced step-like transitions. The former result must indicate that AII amacrine cell number is a polygenic trait arising from multiple genomic loci, while the latter suggests that a few of these genomic loci exert outsized effects relative to the others.

### Variation in AII amacrine cell number maps to quantitative trait loci on Chrs 9, 11, and 19

**FIGURE 2:**
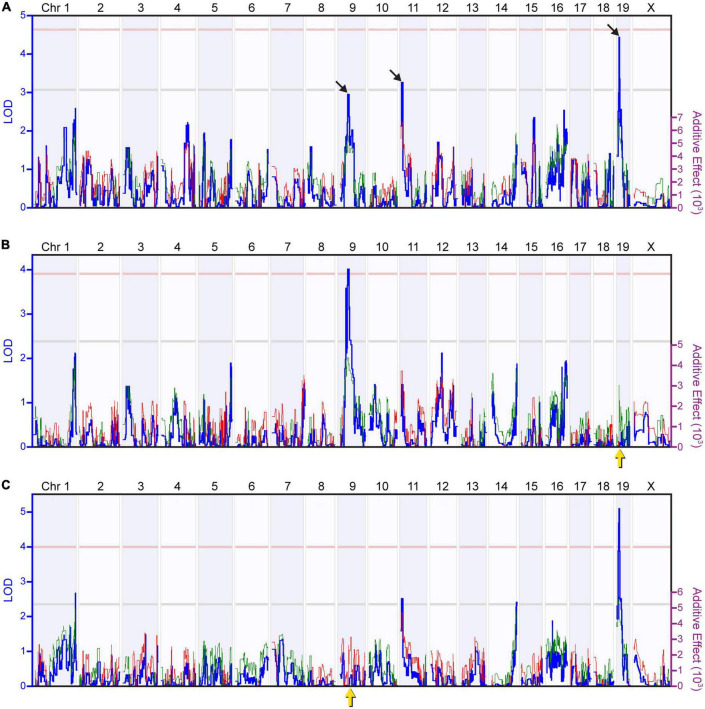
Quantitative trait locus (QTL) mapping identifies discrete genomic loci controlling the number of AII amacrine cells. **(A)** Mapping the variation in AII amacrine cell number revealed three suggestive or near-suggestive genomic loci, on Chrs 9, 11, and 19 (black arrows). The blue trace plots the logarithm of the odds (LOD) score, indicated along the left Y axis. The pink and gray horizontal dashed lines indicate respective significant and suggestive thresholds, respectively, for the LOD score determined through 2,000 permutation tests of the strain data. The red and green traces plot the additive effect of *A* or *B* alleles at each genomic locus upon AII cell number, indicated along the right Y axis. The presence of *A* alleles on Chrs 9 and 19 correlated with an increase in AII amacrine cell number, whereas the presence of *B* alleles on Chr 11 correlated with an increase in AII amacrine cell number. **(B)** Using composite interval mapping to control for the effect of the variation attributed to the QTL on Chr 19 (yellow arrow) raised the LOD score for the Chr 9 locus above the significant threshold. **(C)** Conversely, controlling for the effect of the variation attributed to the QTL on Chr 9 (yellow arrow) raised the LOD score for the Chr 19 locus far above the significant threshold. These two composite interval maps validate the presence of the two QTLs.

By associating the variation in AII amacrine cell number to the variation in parental strain haplotype across the genome of these RI strains, we mapped this variation to three prospective genomic loci, on Chrs 9, 11, and 19 (Figure 2A). Permutation mapping indicated that none of the LOD scores associated with these three loci reached the significant threshold (pink broken line, *p* < 0.05), though two of them crossed the suggestive threshold (gray broken line, *p* < 0.67), and the third nearly reached this level. The estimated additive effects associated with the presence of two *A* alleles at the Chr 9 locus (LOD = 2.96; 46.49 Mb) equaled 11,139 cells, and at the Chr 19 locus (LOD = 4.44; 12.40 Mb) equaled 13,585 cells, while the presence of two *B* alleles at the Chr 11 locus (LOD = 3.27; 11.07 Mb) equaled 13,252 cells. Thus, the countervailing effects of the two haplotypes at these three loci, plus those at other locations that make smaller contributions, must be operating additively to render the parental strains hardly different.

Composite interval mapping can be used to map the remaining variation in cell number after accounting for the variation mapped to a primary locus. We used composite interval mapping to determine whether we might better reveal any undetected genomic loci by controlling for the variation due to these primary loci. When controlling for the variation associated to the locus on Chr 19, we found the locus on Chr 9 to surpass the significant threshold, validating this QTL, while additional suggestive loci on Chrs 1 and 16 were revealed, each with *A* alleles being associated with an increase in trait values (Figure 2B). Likewise, by controlling for the variation associated to the locus on Chr 9, the locus on Chr 19 far surpassed the significant threshold, while an additional suggestive locus on Chr 14 was revealed (Figure 2C), with the latter also indicating an effect of *A* alleles increasing trait values. Controlling for the variation associated to the locus on Chr 11 revealed again the slightly suggestive QTL on Chr 1 (not shown), but no other novel loci were observed. Overall, this exercise in composite interval mapping, while not revealing prominent loci at other locations, confirmed the presence of two large-effect QTL from the three originally identified, on Chr 9 and 19, where the presence of *A* alleles at each locus increased trait values. While the third of the three originally identified loci, the locus on Chr 11, is still detected when controlling for the other two, its magnitude is diminished (Figures 2B, C), and so we have focused our attention on the two significant QTLs on Chrs 9 and 19.

**FIGURE 3:**
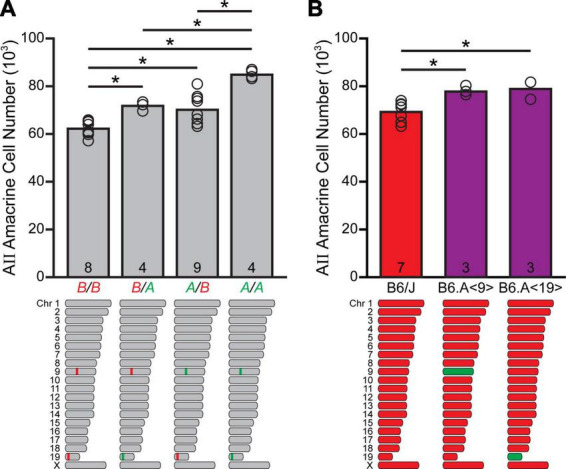
The quantitative trait loci (QTLs) on Chr 9 and Chr 19 are additive in their effects upon AII amacrine cell number. **(A)** The four recombinant inbred (RI) strains harboring the *A* haplotype at both loci have, on average, ∼84,000 AII amacrine cells, whereas the seven RI strains containing the *B* haplotype at both loci have ∼62,000 cells. Strains having *A* at one locus and *B* at the other locus contain intermediate numbers of AII amacrine cells. n = the number of RI strains with each haplotype combination at the respective loci on Chr 9 and 19. **(B)** Chromosome substitution strain mice, bearing the *A* haplotype for Chr 9 or Chr 19 introgressed upon a B6/J genetic background, each contained roughly 9,000–10,000 more AII amacrine cells than did B6/J mice. n = the number of retinas sampled.

That these two loci work in an additive fashion is made apparent by considering their combined effects upon AII amacrine cell number: the four strains containing the *A* haplotype at both loci averaged about 84,000 AII amacrine cells, while the seven with the *B* haplotype at both loci averaged ∼ 62,000 cells, or a difference of ∼22,000 cells (*p* < 0.001; Figure 3A). This is of a magnitude roughly comparable to the additive effects ascribed to the two loci themselves, accounting for ∼75% of the total variation observed across the entire RI strain-set. The remaining strains, having the *A* haplotype at one locus and the *B* haplotype at the other locus, contain intermediate numbers of AII amacrine cells that are not significantly different (*p* = 0.563), while each was significantly different from the groups containing the same haplotype at both loci (*p* < 0.005), albeit with considerable scatter amongst the strains containing the *A* haplotype on Chr 9 and *B* on Chr 19, likely indicative of other participating smaller-effect loci.

### Chromosome substitution strain mice validate QTLs on Chrs 9 and 19

In order to validate independently the presence of gene variants for which *A* alleles increase trait values on these chromosomes, we examined chromosome substitution strain mice. These are inbred strains available from The Jackson Laboratory in which a single chromosome from the donor A/J strain replaces the corresponding chromosome in the host B6/J strain, produced through standard genetic principles, including at least ten sequential backcrosses while genotyping progeny at each generation, followed by intercrossing in order to homozygose the substituted chromosome (Nadeau et al., 2012). Specifically, AII amacrine cell numbers were quantified in mice in which the entire *A* haplotype on Chr 9 (B6.A<9>), or on Chr 19 (B6.A<19>), had been introgressed onto the B6/J genetic background. B6.A<9> mice had an average total of 77,951 cells, while B6.A<19> mice had an average total of 79,085 cells (Figure 3B), these totals being roughly 8,700 and 9,900 more cells than observed in the B6/J strain (*p* = 0.02 and 0.007, respectively). These values are, of course, revealing the net effects of all variant genes on each chromosome, not just the effects associated with those respective genomic loci, and so should not be expected to match precisely those QTL effects. But the magnitude of their effects (each about 30% of the overall variation present across the RI strain-set) reinforces the mapped identification of two QTLs on these chromosomes that each exert outsized effects on the total number of AII amacrine cells. That each of these chromosome substitution strains has greater numbers of AII amacrine cells than the A/J strain, despite their all carrying the *A* haplotype throughout Chrs 9 and 19, must substantiate the presence of other genomic loci (including the Chr 11 locus) where the presence of the *B* haplotype elevates cell number.

### Candidate gene analysis identifies potential causal genes at each locus

**FIGURE 4:**
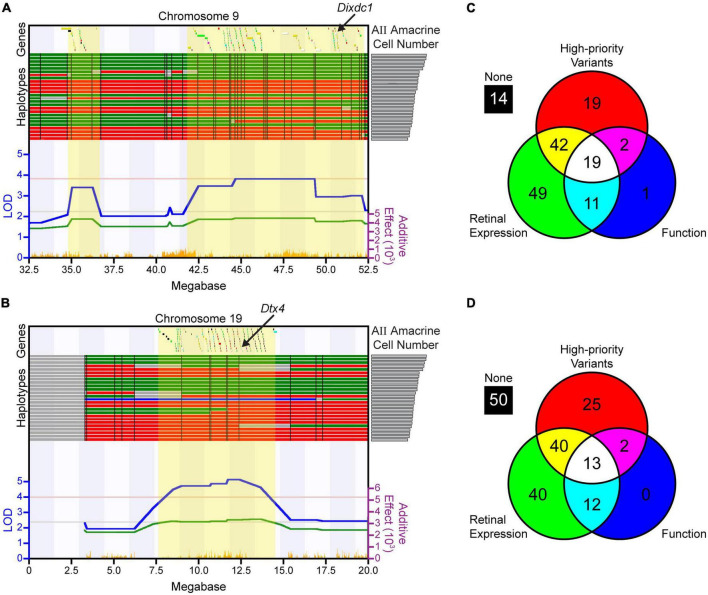
Candidate gene analysis was performed at each quantitative trait locus (QTL). **(A,B)** All of the genes within the respective QTLs on Chr 9 **(A)** and Chr 19 **(B)**, indicated across the top of each map (positions relative to the mm9 assembly), were first interrogated for the presence of sequence variants; those containing no sequence variants were eliminated from further consideration. The remaining genes were assessed for the presence of high-priority variants, retinal expression, and relevant functions as described in the Methods. Genes are color-coded based on whether one, two, or all three of these criteria were met, based on the colors indicated in the Venn diagrams in **(C,D)**. The haplotype map for each strain is shown directly above the QTL map, ranked from the strain with the most AII amacrine cells (top) to the least (bottom), as indicated by the histogram from Figure 1D now shown on the right. Single nucleotide polymorphisms (SNP) density across each region is shown at the bottom in orange. Other conventions as in Figure 2. **(C,D)** The results of the bioinformatic analyses at each QTL are represented by Venn diagrams, revealing that 19 genes at the locus on Chr 9 and 13 genes at the locus on Chr 19 met all three criteria and were assessed further, after which, *Dixdc1* and *Dtx4* were chosen as the top candidate genes. Supplementary Tables 1, 2 list all genes at each QTL, and contain a summary of the bioinformatic analysis for each, while Supplementary Table 3 describes these 32 candidate genes in greater detail.

The QTL on Chr 9 encompasses 157 genes (Figure 4A), while the QTL on Chr 19 contains 182 genes (Figure 4B). For each of these genes, sequence variants were analyzed, retinal expression was assessed, and known functions were identified, as described in the Methods. The analysis for all 339 genes is summarized in Supplementary Tables 1, 2. Nineteen genes at the Chr 9 locus (Figure 4C) and 13 genes at the Chr 19 locus (Figure 4D) were identified as top candidate genes, as they met all three of the following criteria (shown in Supplementary Tables 1, 2): (1) having at least one high-priority sequence variant between the A/J and B6/J genomes, as described in the Methods, (2) exhibiting expression in the retina during development or in adulthood, and (3) having a function that could influence gene regulation, cell proliferation, or apoptosis (Supplementary Table 3). As can be surmised from Supplementary Tables 1, 2, some of these genes in Supplementary Table 3 were regarded as more promising avenues for further exploration, because they had been found to be expressed not only in the adult retina but also during development; others had both potential regulatory as well as functional variants; and some of these genes were known to be associated with genetic pathways previously shown to affect retinal development. For instance, *Cell adhesion molecule 1* (*Cadm1)* is a synaptic cell adhesion molecule expressed by developing cells in the retina, particularly photoreceptors, and participates in the formation of ribbon synapses in the outer plexiform layer (Ribic et al., 2014). It contains several splice region variants as well as several variants directly upstream of the transcriptional start site. *Cell adhesion associated, oncogene regulated (Cdon)*, is a ROBO-related cell surface protein associated with holoprosencephaly and is a receptor in the Hedgehog signaling pathway involved in early eye patterning (Cardozo et al., 2014). It too contains several splice site region variants; additionally, two missense variants discriminate the parental strains. *Zw10 kinetochore protein* (*Zw10*) is a mitotic checkpoint protein that is expressed in the developing retina, and several regulatory variants exist. *Family with sequence similarity 111, member A* (*Fam111a*) is a serine protease that is important for ensuring proper DNA replication and acts as a cell cycle checkpoint protein. Single amino acid mutations in this gene are associated with Kenny-Caffey syndrome in humans, of which microphthalmia is one manifestation (Lang et al., 2021); between the A/J and B6/J parental strains, there are 46 missense variants. Syntaxin 3 (*Stx3*) is part of the SNARE complex and participates in vesicle exocytosis and protein trafficking. It is expressed in developing and mature AII amacrine cells, among other retinal neurons, and removal of *Stx3* from photoreceptors leads to rapid degeneration (Kakakhel et al., 2020).

We began by probing these and other interesting candidates by validating their expression patterns, correlating expression levels with AII amacrine cell number, or evaluating the effects of knockout models (e.g., *in situ* hybridization showed elevated *Cdon* expression in the adult retina within the INL, but analysis of *Cdon*-KO mice revealed no change in the number of AII amacrine cells). As we proceeded, two genes became of particular interest, *Dixdc1* and *Dtx4*, to which we directed increasing attention, and are discussed in detail below.

### *Dtx4* is a promising candidate on Chr 19

**FIGURE 5:**
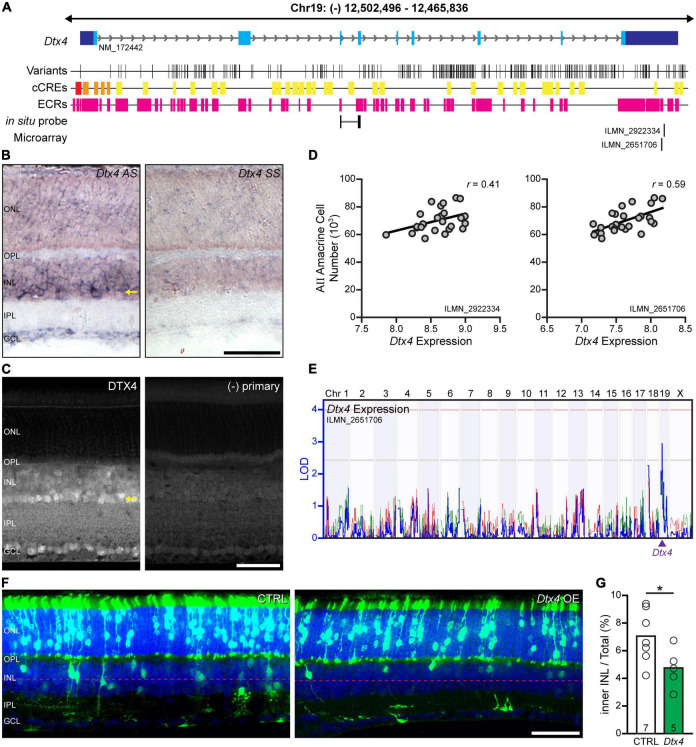
*Dtx4* overexpression decreases amacrine cell number. **(A)** The genomic structure of *Dtx4*, with positions relative to the mm10 assembly and all annotated RefSeq transcripts shown. The *Dtx4* gene has 9 exons and is ∼45 kb in size. The location of each exon is indicated (blue boxes), with the 3′ and 5′ UTRs in dark blue and coding sequence in light blue. The positions of each sequence variant that discriminate the A/J and B6/J genomes are indicated below, along with the location of candidate *cis*-regulatory elements (cCREs: red = promoter-like signature; orange = proximal enhancer-like signature; yellow = distal enhancer-like signature; green = DNase-H3K4me3 sites) annotated by the ENCODE Data Analysis Center, and evolutionarily conserved regions (ECRs). The position of the *in situ* hybridization probe and microarray probes are indicated along the bottom. **(B)** *Dtx4* exhibits heightened expression in the amacrine cell layer (ACL). Expression throughout the retina is present (compare with sense probe; left) but shows greatest expression amongst the amacrine cells in the innermost portion of the INL (arrow). **(C)** Immunofluorescence corroborates the heightened presence of DTX4 in the ACL (arrow). **(D)** Microarray analysis of ocular mRNA from the recombinant inbred (RI) strains in maturity demonstrates a positive correlation with AII amacrine cell number, for both *Dtx4* probes on the array. Values are normalized to mean expression across the entire microarray, with each unit indicating a doubling in expression, with values above 7 being considered above background. **(E)** That variation in *Dtx4* expression across the strains maps a *cis*-eQTL at the *Dtx4* locus for one of the probes, where the *A* haplotype is associated with increased expression. **(F)** Plasmid overexpression of *Dtx4 via* electroporation on P2 yields GFP-positive cells in the ONL and INL at P21. **(G)** *Dtx4* overexpression reduces the frequency of GFP-positive cells in the inner half of the INL. n = the number of transfected retinas in panel **(G)**. Calibration bar in panels **(B,C,F)** = 50 μm.

Of those higher priority candidate genes on Chr 19, *Deltex E3 ubiquitin ligase 4 (Dtx4)*, was considered of particular interest, because of its modulatory role upon Notch signaling (Revici et al., 2022), the latter recognized to participate in the balance between progenitor cell proliferation versus cell cycle exit and differentiation (Kageyama et al., 2009), including within the retina (Rapaport and Dorsky, 1998; Mills and Goldman, 2017). It has been shown from single cell transcriptomic data to be expressed in retinal progenitor cells in embryonic and early postnatal retina (Clark et al., 2019; Balasubramanian et al., 2021), as well as in AII amacrine cells in maturity, along with several other amacrine cell types (Yan et al., 2020). Finally, there exist 309 sequence variants discriminating the B6/J and A/J genomes (Figure 5A), two variants of which were noted as high priority in the initial screen: a structural variant in the fourth intron and a SNP in the 3′UTR, both lie within evolutionarily conserved regions.

### Variation in *Dtx4* across the RI strains correlates with AII amacrine cell number

We confirmed *Dtx4* expression in the mature B6/J retina using *in situ* hybridization, noting heightened expression in the innermost portion of the INL where amacrine cells are positioned (Figure 5B). Immunolabeling of adult B6/J sections with an antibody to DTX4 corroborated the expression pattern seen with *in situ* hybridization, revealing a punctate intracellular pattern of staining that was most obvious in the ACL (Figure 5C). All of the AXB/BXA RI strains have previously been profiled transcriptionally *via* microarray analysis of ocular mRNA (Whitney et al., 2011), which can be used to assess both variation in gene expression across the strains as well as to detect correlations between gene expression and other traits, such as cell number. Two microarray probes measured *Dtx4* transcript levels across the RI strains (IDs ILMN_2922334 and ILMN_2651706), both mapping to the distal 3′UTR of the mRNA (Figure 5A); the output of both probes exhibited a high positive correlation between them (*r* = 0.72). These probes revealed that *Dtx4* expression was variable across the RI strains, and, notably, this variation was positively correlated with the variation in AII amacrine cell number (Figure 5D). Furthermore, the variation in *Dtx4* expression (measured from probe ID LIMN_2651706) mapped a suggestive expression (e)QTL to the location of *Dtx4* on Chr 19, that is, a *cis*-eQTL, revealing the *A* haplotype at this locus to be associated with an increase in *Dtx4* expression (Figure 5E). Given the difference in expression in *Dtx4* across the strains, all 309 variants, including those found in intronic regions, were scrutinized in-depth by cross-referencing their locations with candidate *cis*-regulatory elements (cCREs) identified using the GENCODE database. Several INDELs were found in the promoter region; additionally multiple SNPs and INDELs were identified in putative enhancer sites (Figure 5A). Variation in the expression of *Dtx4*, therefore, is likely caused by one or more of these putative regulatory variants, which in turn may influence the final number of AII amacrine cells in the retina, and thus underlie the QTL on Chr 19.

### *Dtx4* overexpression reduces the frequency of amacrine cells

Since *Dtx4* is present in retinal progenitors as early as E13.5 and continues to be expressed in progenitors through the perinatal period (Clark et al., 2019; Balasubramanian et al., 2021), we sought to assess a functional role for *Dtx4* during development using an overexpression strategy *via* electroporation on the day after birth. Previous studies in our lab (Kautzman et al., 2018) confirm earlier demonstrations that electroporation at this age yields transfection only of cell types that are generated postnatally, including rod photoreceptors, bipolar cells, Müller glia, and some amacrine cells (Matsuda and Cepko, 2004, 2007). Plasmids encoding *gfp* or both *Dtx4* and *gfp* were used to transfect progenitor cells at this time-point; when mice developed to 3 weeks of age, we identified the position of all GFP-positive cells and determined the proportion of them positioned in the ACL. Twelve retinas showed successful GFP expression, including seven control retinas and five *Dtx4-*overexpressing retinas, each group averaging ∼850 transfected cells per retina, the vast majority of cells (75–80%) being positioned in the ONL. Because most AII amacrine cells are generated before birth (Voinescu et al., 2009), their expected frequency within the GFP+ cohort should be small, and so rather than immunolabeling them to distinguish this rare contingent amongst the GFP+ cells, we simply counted all GFP+ cells situated within the ACL (Figure 5F). When those GFP+ cells (specifically, those in the inner half of the INL) were considered as a proportion of that total GFP population in each of these retinas (Figure 5G), a significant reduction was found in the *Dtx4-*overexpressing retinas (*p* = 0.047). Overexpression of this gene during development, therefore, would appear to reduce the production of amacrine cells, likely including the AII amacrine cell because it is one of the later-generated amacrine cell types (Voinescu et al., 2009).

### *Dixdc1* is a promising candidate on Chr 9

*Dix domain containing 1 (Dixdc1)* is a positive regulator of the WNT-signaling pathway (Shiomi et al., 2003; Liu et al., 2011). *Dixdc1* expression during murine development was previously characterized *via* RT-PCR and shown to be expressed as early as E9.5 (Shiomi et al., 2005). Specific tissues were profiled *via in situ* hybridization, revealing expression in the CNS as early as E9.5 (Soma et al., 2006). Within the developing neocortex, *Dixdc1* has been shown to play a role in neuronal proliferation and migration (Singh et al., 2010), and dendritic development and synapse function (Guo et al., 2018; Martin et al., 2018), and multiple sequence-disrupting single nucleotide variants in *DIXDC1* have been associated with mental illness and autism (Martin et al., 2018). Studies in zebrafish demonstrated a reduction in eye size following overexpression of *Dixdc1* during development (Shiomi et al., 2003), while loss of *Dixdc1* in the mouse has been shown to delay retinal angiogenesis (Kim et al., 2022). *In situ* hybridization in the mouse retina revealed expression confined to the inner retina as early as E13.5 (Shiomi et al., 2005), and continuing there through the prenatal period (Soma et al., 2006), when amacrine cells are being generated (Voinescu et al., 2009). As a *Dixdc1* knockout mouse is available, we profiled the retinas of these mice to assess a potential role for *Dixdc1* in the regulation of AII amacrine cell number.

### *Dixdc1*-knockout mice have excess numbers of AII amacrine cells

**FIGURE 6:**
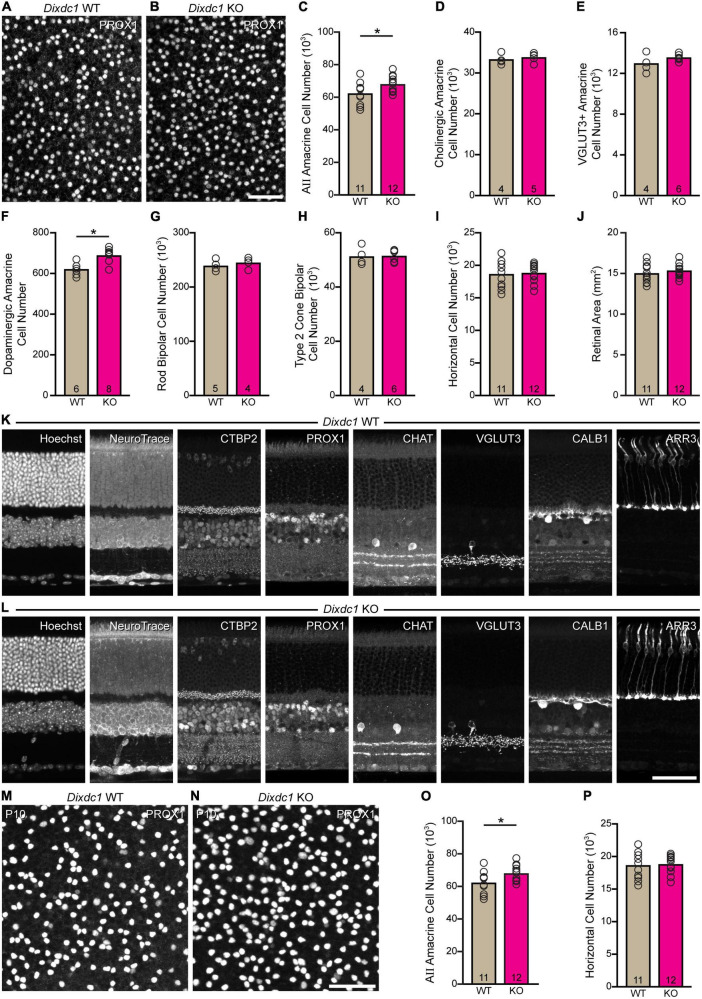
Loss of *Dixdc1* increases AII amacrine cell number. **(A–C)** *Dixdc1* KO retinas have a significant increase in the size of their AII amacrine cell population, by about 10%. **(D–I)** Of six other sampled retinal populations, only the dopaminergic amacrine cells were significantly different, showing a comparable increase in size. **(J)** The areal size of the retina was unaltered in *Dixdc1* KO mice. **(K,L)** Both the cellular and synaptic architecture of the retina were unaffected. **(M–P)** A replication at P10 confirmed a comparable effect upon AII amacrine cell number, while finding no difference in horizontal cell number. n = the number of retinas sampled in panels **(C–J,O,P)**. Calibration bar in panels **(A,B,K–N)** = 50 μm.

AII cell densities were determined in *Dixdc1* KO and littermate WT mice, to estimate total cell number. There was a significant increase in the size of the AII amacrine cell population in *Dixdc1* KO retinas, by nearly 6,000 cells (*p* = 0.024) (Figures 6A–C). We also assessed the breadth of the effect of loss of *Dixdc1* function by determining the size of other neuronal populations in *Dixdc1* KO and littermate WT mice, including cholinergic amacrine cells, VGluT3 amacrine cells, dopaminergic amacrine cells, rod bipolar cells, one type of cone bipolar cell (the Type 2 cone bipolar cell), and horizontal cells (Figures 6D–I). Only the dopaminergic amacrine cell population showed a significant change (*p* = 0.002), being an increase of comparable magnitude to that observed for AII cells, by about 11% (Figure 6F). No change in the overall size of the retina was observed (*p* = 0.413) (Figure 6J), and nor were there any observable abnormalities in the cellular or synaptic architecture of the retina (Figures 6K, L). Finally, we validated this effect upon AII cells by sampling additional *Dixdc1* KO and littermate control (WT) retinas, now at P10, counting the AII amacrine cell population along with one other cell type that did not change in the adult, the horizontal cell population. As in maturity, we found a comparable increase in AII amacrine cells at P10, by 12.5% (*p* < 0.001) (Figures 6M–O), while the horizontal cell population was unchanged (*p* = 0.921) (Figure 6P).

### A missense variant and splice variant discriminate the B6/J from A/J genomes

**FIGURE 7:**
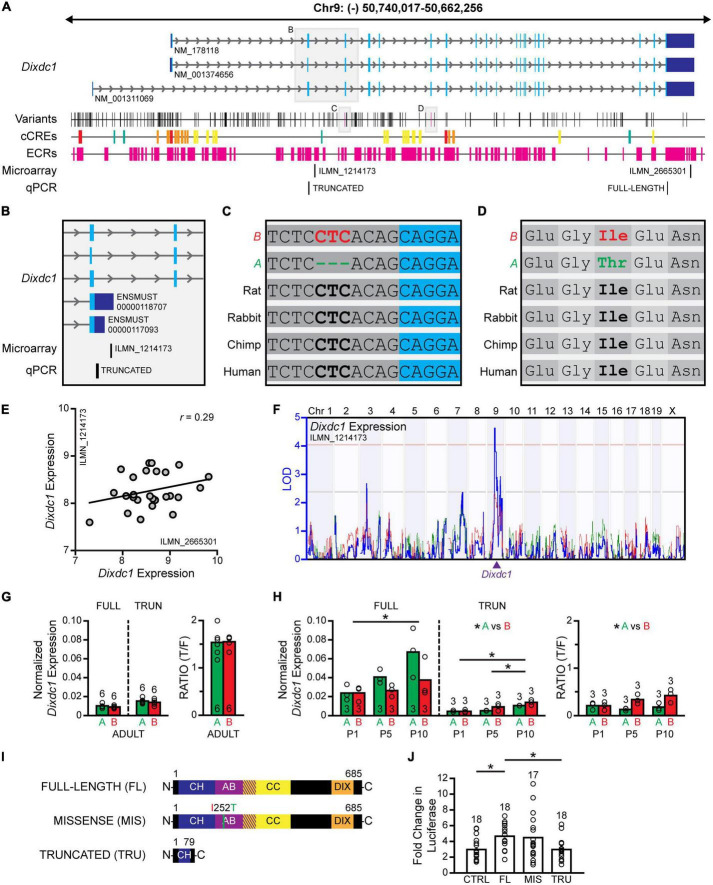
Full-length and truncated *Dixdc1* transcripts are differentially regulated during development. **(A)** The genomic structure of *Dixdc1*, with conventions as in Figure 5A. Three transcripts are annotated in the RefSeq database, each with 20 exons and ∼77 kb in size. A/J and B6/J genomes are discriminated by many sequence variants, including one missense variant and one splice region INDEL of particular interest. The positions of two microarray probes are indicated, one probe recognizing the 3′UTR of the three annotated transcripts, and one probe that is complementary to a portion of the second intron. The positions of the qPCR primers are indicated at the very bottom. **(B)** Magnified region encompassing the second intron. Two transcripts that terminate within this intron were identified in the Ensembl database (release 102), one of which is recognized by the microarray probe. **(C)** The splice variant, a three-nucleotide INDEL, is in the second intron near the splice donor site. **(D)** The missense variant results in an amino acid substitution from nonpolar isoleucine to a polar threonine within the actin binding domain of *Dixdc1*. B6/J carries the evolutionarily conserved sequence in each case. **(E)** Microarray analysis derived from adult whole eye mRNA (normalization described in Figure 5D) confirms *Dixdc1* expression using either the 3′UTR probe or the intronic probe in each RI strain, showing considerable (if largely uncorrelated) variation across strains. **(F)** Variation in the expression of truncated transcripts (ENSMUST00000118707) mapped a *cis*-eQTL, where the presence of the *B* haplotype at this locus was correlated with increased expression. **(G)** qPCR analysis reveals the truncated transcripts to be the dominant form in maturity, showing comparable levels of each transcript in both strains. **(H)** During postnatal development, the full-length transcripts are more prevalent, with expression of both transcripts increasing significantly as a function of age. Strain differences in expression were observed at later ages, yielding significant differences in the proportion of truncated to full-length transcripts, with B6/J retinas exhibiting a larger proportion than A/J retinas. **(I)** A luciferase expression assay compared the effectiveness of a full-length DIXDC1 isoform (NP_835219; translated from NM_001374656), an identical DIXDC1 isoform but now containing the missense mutation, and a truncated DIXDC1 isoform similar to that expected to be translated from the truncated transcripts, upon DVL2-mediated β-catenin signaling. Functional domains were determined from UniProt (uniport.org) annotations; CH, calponin homology domain; AB, actin binding domain; CC, coiled coil domain; DIX, DIX domain. **(J)** The missense isoform showed no functional effect of the mutation, augmenting β-catenin signaling to similar levels as full-length DIXDC1, while the truncated isoform was ineffective at modulating β-catenin signaling in either direction, with luciferase expression levels identical to controls. n = the number of adult retinas in panel **(F)**, the number of pooled samples from postnatal retinas in panel **(G)**, and the number of wells in panel **(H)**.

The RefSeq database lists three annotated mRNA transcripts, each coding for the full-length DIXDC1 protein (Figure 7A), that is, isoforms which contain all of the known functional domains, including a calponin homology domain, an actin binding domain, two coiled coil domains, and a DIX domain (Shiomi et al., 2005; Wang et al., 2006). Other isoforms are known to arise from the use of multiple promoters and alternative splicing, coding for proteins that lack various lengths of the N-terminal region (Shiomi et al., 2005), and thus altering DIXDC1 function. Amongst the sequence variants in *Dixdc1* discriminating the B6/J from A/J genomes, two particularly interesting variants were scrutinized, including one splice-region disrupting INDEL (Figure 7C) and one missense variant (Figure 7D). The missense variant results in a change from a nonpolar isoleucine to a polar threonine within the actin-binding domain of *Dixdc1* (Figure 7D). The splice region sequence variant, being a three nucleotide INDEL, was of interest as it lies in the polypyrimidine tract at the 3′ end of the second intron (Figure 7C); the location of this INDEL close to the donor splice site suggests that it may influence the functioning of the splicing machinery during mRNA processing. For both sequence variants, the *B* haplotype contained the evolutionarily conserved sequence (Figures 7C, D).

### B6/J and A/J mouse eyes express full-length and truncated *Dixdc1* transcripts

As indicated above, all the RI strains have been profiled transcriptionally *via* microarray analysis (Whitney et al., 2011). The AXB/BXA mouse whole eye mRNA expression database includes two probes for *Dixdc1* (Figure 7A), one of which recognizes the 3′UTR common to each of the annotated transcripts (ILMN_2665301); the other is complementary to a sequence within the second intron of these transcripts (ILMN_1214173). Yet both probes exhibited expression levels that were higher than background (Figure 7E), indicating that the latter probe must be recognizing a novel transcript or transcripts that contain all or part of this intron. Searching various databases revealed that, indeed, two protein coding *Dixdc1* transcripts are expected to terminate within this very intron (Figure 7B). These “truncated” transcripts contain a STOP codon in this intronic sequence, so translation of these transcripts would be expected to yield a truncated protein product lacking most of the C-terminal domains of DIXDC1. Both probes exhibit expression levels that vary across the parental and RI strains of mice but show minimal covariation with one another (Figure 7E). Furthermore, while the variation in the expression of full-length transcripts across the strains does not map to any genomic locus, variation in the expression of truncated transcripts *does* map a significant *cis*-eQTL on Chr 9 at the *Dixdc1* locus (Figure 7F), implicating a sequence variant in those strains carrying the *A* haplotype at this locus in lowering the level of truncated transcripts, increasing the potential candidacy of the splice region INDEL. Unlike *Dtx4*, adult ocular expression of *Dixdc1* was not correlated with AII amacrine cell number, whether measuring the expression of full-length transcripts (*r* = −0.15) or truncated transcripts (*r* = 0.03).

### B6/J and A/J retinas differ in their relative expression of the truncated and full-length *Dixdc1* transcripts during development

To compare expression of the different *Dixdc1* transcripts across development, we designed two sets of primers to quantify levels of full-length transcripts versus those that are truncated in the second intron, using qPCR (Figure 7A). We first compared adult retinal mRNA, finding that both strains expressed similar amounts of the full-length (*p* = 0.27) as well as truncated (*p* = 0.42) transcripts (Figure 7G), yielding comparable ratios of truncated to full-length transcripts (*p* = 0.92), with the truncated transcripts being more prevalent.

We subsequently examined mRNA levels during postnatal development, at P1, P5, and P10, during the period when AII amacrine cells are concluding their neurogenesis and commencing their differentiation (Voinescu et al., 2009; Gamlin et al., 2020). There, we observed a progressive increase in both sets of *Dixdc1* transcripts during the first 10 postnatal days, with a two-way ANOVA detecting a significant main effect of age (Figure 7H; full-length, *p* = 0.020; truncated, *p* < 0.001). A significant main effect of strain was also detected for the truncated transcripts (*p* = 0.027), with the B6/J strain exhibiting higher expression than the A/J strain; although no significant effect of strain was observed for the full-length transcripts (*p* = 0.062), the A/J strain appeared to have a higher expression than the B6/J strain at the later postnatal ages (Figure 7H). These opposing effects seen between the strains resulted in a ratio of truncated to full-length transcripts that also differed, yielding a significant main effect of strain (*p* = 0.005) with B6/J having a higher relative abundance of truncated transcripts. In sum, the efficiency of this splicing event may be altered transiently after birth due to the presence of the splice region INDEL; as development proceeds beyond P10, the abundance of full-length transcripts decreases and the truncated transcripts become the dominant form, and differences between the strains in the ratio of these transcripts are no longer present (Figure 7G).

### The truncated DIXDC1 protein does not modulate β-catenin signaling

The shortened *Dixdc1* transcripts have a STOP codon within the retained section of the second intron, yielding a truncated protein product lacking most of the C-terminus. We subsequently designed a luciferase assay to determine if a similarly truncated protein product would have any effect on β-catenin signaling; additionally, we examined any potential functional consequence of the missense variant mentioned above. HEK cells were co-transfected with a β-catenin luciferase reporter plasmid and expression plasmids that either did not encode a protein product (control) or expressed one of three different *Dixdc1* transcripts coding for different DIXDC1 isoforms (Figure 7I): a full-length DIXDC1 (full-length), a full-length DIXDC1 that contains the amino acid substitution found in the *A* sequence (missense), and a DIXDC1 isoform lacking most of the C-terminus (truncated). Additionally, half the wells were co-transfected with a plasmid encoding *Disheveled2* (*Dvl2*), an effective activator of WNT signaling. For each DIXDC1 condition (control, full-length, truncated, and missense), the luciferase expression in the presence of DVL2 was normalized to the expression observed absent DVL2 (i.e., with no activation of WNT signaling), to compare the fold change in β-catenin signaling upon WNT activation in the presence of each DIXDC1 isoform.

A one-way ANOVA confirmed significant differences across the four conditions (*p* = 0.008). Tukey’s *post-hoc* testing confirmed that the full-length DIXDC1 isoform significantly increased DVL2-induced β-catenin signaling relative to controls lacking any *Dixdc1* transcript (*p* = 0.04), as expected given its role as a positive regulator of WNT signaling (Figure 7J). The substitution of the *A* missense mutation in that full-length DIXDC1 isoform yielded comparable DVL2-induced β-catenin signaling (*p* = 0.99), suggesting that its presence does not alter DIXDC1 function (Figure 7J). Substituting the truncated DIXDC1 isoform in place of the full-length isoform yielded levels of DVL2-induced β-catenin signaling that were significantly lower (*p* = 0.04), being comparable to those achieved in the control condition lacking any *Dixdc1* transcript (*p* = 1.00) (Figure 7J). We conclude, consequently, that any truncated protein that is produced *in vivo* should be ineffective in either promoting or reducing WNT-β-catenin signaling, but whether it might possess any other functional significance, such as acting as a dominant negative isoform, remains to be determined.

## Discussion

The number of neurons in a population is not a fixed trait for a given species. Rather, cell number is controlled by a delicate balance of developmental processes regulating their production, differentiation and survival, each modulated by genetic determinants that themselves vary due to sequence variants affecting their function or expression. The present study has investigated the large variation in the number of one such population of neurons, the AII amacrine cells. Within-strain variation is meager relative to the between-strain variation, indicating the heritability of this trait, and that latter variation is graded, indicating it must be polygenic in origin. We mapped that variation to three prospective QTLs on Chrs 9, 11, and 19, and confirmed two of these QTLs on Chrs 9 and 19 through composite interval mapping. We independently validated the presence of causal genomic variants on Chrs 9 and 19 using consomic chromosome substitution strains.

Following extensive bioinformatic analyses, we winnowed down the genes present at these two QTLs to identify high-priority candidates worthy of further investigation, scrutinizing two such candidates in greatest detail, *Dtx4* and *Dixdc1*. We found elevated expression of *Dtx4* within the ACL in the mature B6/J retina, when *Dtx4* expression levels were found to correlate with the variation in AII amacrine cell number across the RI strains. That expression in maturity across the strains mapped a *cis*-eQTL indicative of causal regulatory variants present in *Dtx4*, and sequence comparisons confirm *Dtx4* is amply endowed with SNPs and INDELs in prospective regulatory regions. We electroporated *Dtx4*-encoding plasmids in an effort to overexpress it on the day after birth, recognizing this age to be near the end of the neurogenetic window for AII amacrine cell production (Voinescu et al., 2009), yet we still achieved a significant reduction in the proportion of amacrine cells produced amongst the cohort of transfected cells. Overexpression of *Dtx4* at this age, consequently, decreases the frequency of amacrine cells in the INL, interpreted to reflect a reduction in the number produced due to positive regulation of Notch signaling, suppressing pro-neural gene activity and maintaining cells in a proliferative state (Revici et al., 2022). This result, however, runs contrary to the positive correlation between *Dtx4* expression levels and AII amacrine cell numbers in maturity (Figure 5D), but it is possible that endogenous expression of *Dtx4* is more influential earlier in development than in our overexpression study, altering proliferation in such a manner to increase amacrine cell number. The presence of a regulatory variant or variants likely contributes to the modulation of *Dtx4* that underlies the *cis*-eQTL, in turn contributing to the variation in AII amacrine cell number seen across the strains.

That *Dixdc1* plays a role in modulating AII amacrine cell number was confirmed by examining its loss of function: *Dixdc1* KO retinas contained elevated numbers of AII amacrine cells, by about 6,000 cells, an increase of about 10%. This is slightly more than half of the QTL effect on Chr 9, suggesting that there is another additional variant gene or genes at this QTL (Indeed, a search for variant genes controlling the number of dopaminergic amacrine cells at a QTL on Chr 7 revealed the presence of three different genes with countervailing effects upon dopaminergic cell number within that interval; Whitney et al., 2009). Retinal expression of *Dixdc1* is present prenatally (Shiomi et al., 2005; Soma et al., 2006), during the period of AII amacrine cell genesis (Voinescu et al., 2009), as well as postnatally (shown here), when AII amacrine cells differentiate (Gamlin et al., 2020). Full-length *Dixdc1* transcripts as well as truncated *Dixdc1* transcripts are expressed in developing mouse retina, and strain differences in the ratio of full-length to truncated *Dixdc1* transcripts were detected postnatally, largely after the neurogenetic period, suggesting that the role played by *Dixdc1* in modulating cell number occurs after their production. AII amacrine cell numbers are overproduced during development, evidenced by their 33% increase in the absence of the pro-apoptotic *Bax* gene (Keeley et al., 2022), and so the degree of naturally-occurring cell death may be modulated though *Dixdc1* driving successful differentiation. Indeed, the increase in AII cell number observed in *Dixdc1* KO mice was already achieved by P10, narrowing the window of DIXDC1 action to this postnatal period.

The two most promising *Dixdc1* sequence variants discriminating the parental genomes, a missense variant and the splice-site INDEL, were investigated in further detail *in vitro* using a β-catenin reporter luciferase assay. The full-length DIXDC1 isoform possessing the amino acid substitution was found to activate β-catenin similarly to the canonical full-length isoform, leading us to dismiss its role *in vivo*. Instead, we addressed whether the truncated isoform might have biological activity in the same assay, finding it neither augmented nor attenuated DVL2-mediated activation of β-catenin signaling; this is perhaps unsurprising given this isoform lacks most of the functional domains, including the DIX domain. As such, we infer that any effect produced by the decline in the efficiency of splicing caused by this splice-region disrupting INDEL is likely due to the reduction in functional (full-length) DIXDC1, in turn affecting AII amacrine cell number. Because the presence of the *B* haplotype at the Chr 9 QTL is correlated with lower numbers of AII amacrine cells (Figures 3A, 4A) and higher expression of the truncated transcripts (Figure 7F), we expect that an increase in non-functional over functional DIXDC1 isoforms would lead to fewer AII amacrine cells. Yet the effect of knocking out *Dixdc1* function entirely was shown to directly elevate, not reduce, AII cell number. Given the prevalence of the truncated over the full-length transcripts in maturity, these results may intimate a novel function of the truncated DIXDC1 protein independent of the WNT-signaling pathway that itself may modulate AII amacrine cell number.

## Data availability statement

The datasets presented in this study can be found in online repositories. The names of the repository/repositories and accession number(s) can be found below: www.genenetwork.org, 10179, GN210.

## Ethics statement

The use of animals in this study was reviewed and approved by the Institutional Animal Care and Use Committee at the University of California, Santa Barbara, and in accord with the NIH Guide for the Care and Use of Laboratory Animals.

## Author contributions

BK, BR, and PK designed the experiments, interpreted the data, and wrote the manuscript. BK, SL, AK, and PK performed the experiments. BK, RB, RP, and PK conducted the data analysis. All authors contributed to the article and approved the submitted version.

## Footnotes

1
https://www.genenetwork.org

2
https://www.sanger.ac.uk/data/mouse-genomes-project

3
https://www.uniprot.org

4
https://ecrbrowser.dcode.org

5
https://www.ncbi.nlm.nih.gov/gene

6
http://david.ncifcrf.gov

7
http://cepko.med.harvard.edu

8
http://nebasechanger.neb.com
