# Supplementary material for: Quantitative trait loci on chromosomes 9 and 19 modulate AII amacrine cell number in the mouse retina
Source: Front Neurosci. 2023 Feb 2;17:1078168. doi: 10.3389/fnins.2023.1078168 (PMC9932814; doi:10.3389/fnins.2023.1078168)
Supplement: Supplementary file 1 [file Table_1.pdf]

Supplementary Table 1: Candidate Gene Analysis of Chr 9 QTL

	High Priority Variants		Expression		Function			Score		
Gene Symbol	Functional	Regulatory	Development	Adult	Gene Regulation	Apoptosis	Proliferation	Variants	Expression	Function
Kirrel3	NO	YES	NO	YES	NO	NO	NO	YES	YES	NO
Kirrel3os	NO	NO	NO	NO	NO	NO	NO	NO	NO	NO
St3gal4	YES	YES	YES	YES	NO	NO	NO	YES	YES	NO
4930581F22Rik	NO	NO	YES	YES	NO	NO	NO	NO	YES	NO
Dcps	NO	YES	YES	YES	YES	NO	NO	YES	YES	YES
Tirap	NO	YES	YES	YES	YES	NO	YES	YES	YES	YES
Foxred1	NO	YES	YES	YES	NO	NO	NO	YES	YES	NO
Srpr	YES	YES	YES	YES	NO	NO	NO	YES	YES	NO
Rpusd4	NO	YES	YES	YES	NO	NO	NO	YES	YES	NO
4933422A05Rik	NO	YES	NO	NO	NO	NO	NO	YES	NO	NO
Cdon	YES	YES	YES	YES	YES	NO	YES	YES	YES	YES
Ddx25	NO	NO	YES	YES	NO	NO	NO	NO	YES	NO
Pus3	NO	NO	YES	YES	NO	NO	NO	NO	YES	NO
Hyls1	NO	YES	NO	YES	NO	NO	NO	YES	YES	NO
Pate4	NO	NO	YES	NO	NO	NO	NO	NO	YES	NO
Pate2	YES	YES	NO	NO	NO	NO	NO	YES	NO	NO
Hspb2	NO	NO	NO	NO	NO	NO	NO	NO	NO	NO
D730048I06Rik	NO	NO	YES	NO	NO	NO	NO	NO	YES	NO
9230110F15Rik	NO	NO	NO	NO	NO	NO	NO	NO	NO	NO
9230113P08Rik	NO	YES	NO	NO	NO	NO	NO	YES	NO	NO
Gm5615	NO	NO	NO	NO	NO	NO	NO	NO	NO	NO
Sorl1	YES	YES	YES	YES	NO	NO	NO	YES	YES	NO
4930546K05Rik	NO	NO	NO	NO	NO	NO	NO	NO	NO	NO
Sc5d	YES	NO	YES	YES	NO	NO	NO	YES	YES	NO
Tecta	YES	YES	NO	YES	NO	NO	NO	YES	YES	NO
Tbcel	NO	NO	NO	YES	NO	NO	NO	NO	YES	NO
Grik4	NO	NO	NO	YES	NO	NO	NO	NO	YES	NO
Arhgef12	NO	NO	YES	YES	NO	NO	NO	NO	YES	NO
Tlcd5	YES	NO	NO	NO	NO	NO	NO	YES	NO	NO
Pou2f3	NO	YES	NO	NO	YES	NO	NO	YES	NO	YES
Oaf	YES	NO	NO	YES	NO	NO	NO	YES	YES	NO
D630033O11Rik	NO	NO	NO	NO	NO	NO	NO	NO	NO	NO
Trim29	YES	NO	NO	YES	YES	NO	NO	YES	YES	YES
Nectin1	YES	NO	NO	NO	NO	NO	NO	YES	NO	NO
Thy1	NO	YES	YES	YES	NO	NO	NO	YES	YES	NO
Rnf26	NO?	NO	YES	YES	NO	NO	NO	NO	YES	NO
Usp2	NO	NO	YES	YES	YES	NO	YES	NO	YES	YES
Mfrp	NO	NO	NO	YES	NO	NO	NO	NO	YES	NO
C1qtnf5	NO	NO	NO	YES	NO	NO	NO	NO	YES	NO
Mcam	NO	NO	YES	YES	NO	NO	NO	NO	YES	NO
Cbl	NO	NO	YES	YES	NO	YES	NO	NO	YES	YES
Ccdc153	NO	NO	NO	NO	NO	NO	NO	NO	NO	NO
Pdzd3	NO	NO	NO	NO	NO	NO	NO	NO	NO	NO
Nlrx1	NO	NO	NO	YES	NO	NO	NO	NO	YES	NO
Abcg4	NO	YES	YES	YES	NO	NO	NO	YES	YES	NO
Mizf	NO	NO	NO	YES	NO	NO	NO	NO	YES	NO
Tmem24	NO	NO	YES	YES	NO	NO	NO	NO	YES	NO
Dpagt1	NO	NO	YES	YES	NO	NO	NO	NO	YES	NO
H2afx	NO	NO	NO	YES	NO	NO	NO	NO	YES	NO
Hmbs	NO	NO	YES	YES	NO	NO	NO	NO	YES	NO
Vps11	NO	NO	NO	YES	NO	NO	NO	NO	YES	NO
Hyou1	NO	NO	YES	YES	NO	YES	NO	NO	YES	YES
Slc37a4	NO	NO	YES	YES	NO	NO	NO	NO	YES	NO
Trappc4	YES	NO	YES	YES	NO	NO	NO	YES	YES	NO
Rps25	NO	NO	YES	YES	NO	NO	NO	NO	YES	NO
Ccdc84	NO	NO	NO	NO	NO	NO	NO	NO	NO	NO
Foxr1	NO	NO	NO	NO	YES	NO	NO	NO	NO	YES
Upk2	NO	YES	NO	YES	NO	NO	NO	YES	YES	NO
Bcl9l	NO	NO	YES	YES	YES	NO	NO	NO	YES	YES
Cxcr5	YES	YES	NO	NO	NO	NO	NO	YES	NO	NO
Ddx6	NO	NO	YES	YES	NO	NO	NO	NO	YES	NO
BC049987	NO	NO	YES	NO	NO	NO	NO	NO	YES	NO
Treh	NO	YES	NO	NO	NO	NO	NO	YES	NO	NO
Phldb1	YES	NO	YES	YES	NO	NO	NO	YES	YES	NO
Arcn1	NO	NO	YES	YES	NO	NO	NO	NO	YES	NO
Ift46	NO	NO	YES	YES	NO	NO	NO	NO	YES	NO
Tmem25	NO	NO	YES	YES	NO	NO	NO	NO	YES	NO
Ttc36	NO	NO	NO	YES	NO	NO	NO	NO	YES	NO
Kmt2a	NO	YES	YES	YES	YES	NO	YES	YES	YES	YES
Atp5l	NO	NO	YES	YES	NO	NO	NO	NO	YES	NO
Ube4a	YES	YES	YES	YES	NO	NO	NO	YES	YES	NO
Cd3g	NO	NO	NO	NO	NO	NO	NO	NO	NO	NO
Cd3d	NO	YES	NO	YES	NO	NO	NO	YES	YES	NO
Cd3e	YES	YES	NO	NO	NO	YES	NO	YES	NO	YES
Mpzl2	NO	NO	NO	YES	NO	NO	NO	NO	YES	NO
Mpzl3	NO	NO	YES	YES	NO	NO	NO	NO	YES	NO
Amica1	NO	NO	NO	YES	NO	NO	NO	NO	YES	NO
Scn2b	NO	YES	NO	YES	NO	NO	NO	YES	YES	NO
Scn4b	NO	YES	NO	YES	NO	NO	NO	YES	YES	NO
Tmprss4	NO	YES	NO	YES	NO	NO	NO	YES	YES	NO
BC049352	NO	YES	NO	NO	NO	NO	NO	YES	NO	NO
Il10ra	NO	NO	NO	YES	NO	NO	NO	NO	YES	NO
1700003G13Rik	NO	YES	NO	NO	NO	NO	NO	YES	NO	NO
Tmprss13	YES	YES	NO	YES	NO	NO	NO	YES	YES	NO
Fxyd6	YES	NO	YES	YES	NO	NO	NO	YES	YES	NO
Fxyd2	NO	YES	YES	YES	NO	NO	YES	YES	YES	YES
4833428L15Rik	NO	YES	NO	NO	NO	NO	NO	YES	NO	NO
Dscaml1	YES	NO	YES	YES	NO	NO	NO	YES	YES	NO
Cep164	NO	NO	YES	YES	NO	NO	YES	NO	YES	YES
Bace1	NO	NO	YES	YES	NO	YES	NO	NO	YES	YES
Rnf214	NO	NO	YES	YES	NO	NO	NO	NO	YES	NO
Pcsk7	NO	NO	YES	YES	NO	NO	NO	NO	YES	NO
Tagln	NO	NO	YES	YES	NO	NO	NO	NO	YES	NO

Sidt2	NO	NO	YES	YES	NO	NO	NO	NO	YES	NO
Pafah1b2	NO	NO	YES	YES	NO	NO	NO	NO	YES	NO
Sik3	YES	YES	YES	YES	NO	NO	NO	YES	YES	NO
Apoa1	YES	YES	YES	YES	NO	NO	NO	YES	YES	NO
Apoc3	YES	YES	NO	NO	NO	NO	NO	YES	NO	NO
Apoa4	NO	NO	YES	YES	NO	NO	NO	NO	YES	NO
Apoa5	NO	NO	YES	NO	NO	NO	NO	NO	YES	NO
Zpr1	NO	NO	NO	YES	YES	YES	YES	NO	YES	YES
Bud13	NO	NO	YES	YES	YES	NO	NO	NO	YES	YES
4931429L15Rik	NO	NO	NO	NO	NO	NO	NO	NO	NO	NO
2900052N01Rik	NO	YES	YES	NO	NO	NO	NO	YES	YES	NO
Gm4791	YES	YES	NO	NO	NO	NO	NO	YES	NO	NO
Cadm1	YES	YES	YES	YES	NO	YES	NO	YES	YES	YES
Nxpe4	YES	YES	YES	YES	NO	NO	NO	YES	YES	NO
Nxpe2	YES	YES	YES	NO	NO	NO	NO	YES	YES	NO
Rexo2	NO	YES	NO	YES	NO	NO	NO	YES	YES	NO
Rbm7	NO	YES	YES	YES	YES	NO	NO	YES	YES	YES
Gm5617	NO	YES	NO	YES	NO	NO	NO	YES	YES	NO
Nnmt	YES	YES	NO	YES	NO	NO	NO	YES	YES	NO
Zbtb16	NO	YES	YES	YES	YES	YES	YES	YES	YES	YES
Htr3b	NO	YES	NO	NO	NO	NO	NO	YES	NO	NO
Usp28	NO	YES	YES	YES	NO	YES	YES	YES	YES	YES
Cldn25-ps	NO	NO	NO	YES	NO	NO	NO	NO	YES	NO
Zw10	YES	YES	YES	YES	NO	NO	YES	YES	YES	YES
Tmprss5	NO	YES	NO	YES	NO	NO	NO	YES	YES	NO
Gm4894	YES	NO	NO	NO	NO	NO	NO	YES	NO	NO
Drd2	NO	YES	NO	YES	YES	NO	YES	YES	YES	YES
Ankk1	YES	YES	NO	YES	NO	NO	YES	YES	YES	YES
Ttc12	YES	YES	YES	YES	NO	NO	NO	YES	YES	NO
Ncam1	NO	YES	YES	YES	NO	NO	YES	YES	YES	YES
2310003N18Rik	NO	YES	NO	NO	NO	NO	NO	YES	NO	NO
Plet1os	NO	YES	NO	NO	NO	NO	NO	YES	NO	NO
Plet1	NO	NO	NO	YES	NO	NO	NO	NO	YES	NO
Pts	NO	NO	YES	YES	NO	NO	NO	NO	YES	NO
Bco2	NO	NO	NO	YES	NO	NO	NO	NO	YES	NO
Il18	NO	NO	NO	YES	YES	YES	YES	NO	YES	YES
Tex12	NO	NO	YES	NO	NO	NO	NO	NO	YES	NO
Sdhd	NO	NO	YES	YES	NO	NO	NO	NO	YES	NO
Timm8b	NO	NO	YES	YES	NO	NO	NO	NO	YES	NO
AU019823	NO	YES	YES	YES	NO	NO	NO	YES	YES	NO
Pih1d2	NO	NO	NO	NO	NO	NO	NO	NO	NO	NO
Dlat	NO	YES	YES	YES	NO	NO	NO	YES	YES	NO
Dixdc1	YES	YES	YES	YES	NO	NO	YES	YES	YES	YES
2310030G06Rik	YES	YES	YES	YES	NO	NO	NO	YES	YES	NO
Htr3a	NO	YES	NO	YES	NO	NO	NO	YES	YES	NO
Cryab	NO	YES	YES	YES	YES	YES	NO	YES	YES	YES
1110032A03Rik	YES	YES	YES	YES	NO	NO	NO	YES	YES	NO
Fdxacb1	YES	YES	NO	NO	NO	NO	NO	YES	YES	NO
Alg9	NO	YES	YES	YES	NO	NO	NO	YES	YES	NO
Ppp2r1b	NO	YES	YES	YES	NO	YES	NO	YES	YES	YES
Sik2	NO	NO	NO	YES	NO	NO	NO	NO	YES	NO
Layn	NO	NO	NO	NO	NO	NO	NO	NO	NO	NO
4833427G06Rik	NO	YES	NO	YES	NO	NO	NO	YES	YES	NO
Btg4	NO	NO	YES	YES	NO	NO	YES	NO	YES	YES
Pou2af1	NO	YES	YES	NO	YES	NO	NO	YES	YES	YES
Gm684	NO	NO	NO	YES	NO	NO	NO	NO	YES	NO
1810046K07Rik	YES	NO	NO	NO	NO	NO	NO	YES	NO	NO
Arhgap20	NO	YES	NO	YES	NO	NO	NO	YES	YES	NO
4933407I05Rik	NO	YES	NO	NO	NO	NO	NO	YES	NO	NO
Gm6980	NO	NO	NO	NO	NO	NO	NO	NO	NO	NO
Fdx1	YES	NO	YES	YES	NO	NO	NO	YES	YES	NO
Gm6981	NO	YES	NO	NO	NO	NO	NO	YES	NO	NO
Rdx	YES	YES	YES	YES	NO	NO	YES	YES	YES	YES
Zc3h12c	NO	NO	YES	NO	NO	NO	NO	NO	YES	NO
