# Supplementary material for: Quantitative trait loci on chromosomes 9 and 19 modulate AII amacrine cell number in the mouse retina
Source: Front Neurosci. 2023 Feb 2;17:1078168. doi: 10.3389/fnins.2023.1078168 (PMC9932814; doi:10.3389/fnins.2023.1078168)
Supplement: Supplementary file 2 [file Table_2.pdf]

Supplementary Table 2: Candidate Gene Analysis of Chr 19 QTL

	High Priority Variants		Expression		Function			Score		
Gene Symbol	Functional	Regulatory	Development	Adult	Gene Regulation	Apoptosis	Proliferation	Variants	Expression	Function
Hrasls5	NO	NO	NO	YES	NO	NO	NO	NO	YES	NO
Slc22a19	NO	NO	YES	YES	NO	NO	NO	NO	YES	NO
Slc22a27	NO	NO	NO	NO	NO	NO	NO	NO	NO	NO
Slc22a28	NO	NO	NO	NO	NO	NO	NO	NO	NO	NO
Slc22a29	NO	NO	NO	NO	NO	NO	NO	NO	NO	NO
Slc22a30	NO	NO	YES	YES	NO	NO	NO	NO	YES	NO
Slc22a8	NO	NO	YES	YES	NO	NO	NO	NO	YES	NO
Slc22a6	NO	NO	YES	NO	NO	NO	NO	NO	YES	NO
9830166K06Rik	NO	NO	NO	NO	NO	NO	NO	NO	NO	NO
Chrm1	NO	NO	YES	YES	NO	NO	NO	NO	YES	NO
Slc3a2	NO	NO	YES	YES	NO	NO	NO	NO	YES	NO
Snhg1	NO	NO	YES	NO	NO	NO	NO	NO	YES	NO
Wdr74	NO	NO	YES	YES	NO	NO	NO	NO	YES	NO
1700092M07Rik	NO	NO	YES	NO	NO	NO	NO	NO	YES	NO
Stx5a	NO	NO	YES	YES	NO	NO	NO	NO	YES	NO
Nxf1	NO	NO	YES	YES	YES	NO	NO	NO	YES	YES
Tmem223	NO	NO	YES	YES	NO	NO	NO	NO	YES	NO
Tmem179b	NO	NO	YES	YES	NO	NO	NO	NO	YES	NO
Taf6l	NO	NO	NO	YES	YES	NO	NO	NO	YES	YES
Gm2518	NO	NO	NO	NO	NO	NO	NO	NO	NO	NO
Polr2g	NO	NO	YES	YES	YES	YES	NO	NO	YES	YES
Zbtb3	YES	YES	NO	YES	YES	NO	NO	YES	YES	YES
Ttc9c	YES	YES	YES	YES	NO	NO	NO	YES	YES	NO
Hnrnpul2	YES	NO	NO	YES	NO	NO	NO	YES	YES	NO
Gng3	NO	NO	YES	YES	NO	NO	NO	NO	YES	NO
Bscl2	NO	YES	YES	YES	NO	NO	NO	YES	YES	NO
Lrrn4cl	NO	NO	YES	YES	NO	NO	NO	NO	YES	NO
Ubxn1	NO	YES	YES	YES	NO	NO	NO	YES	YES	NO
5730408K05Rik	NO	YES	YES	YES	NO	NO	NO	YES	YES	NO
1810009A15Rik	NO	YES	YES	YES	NO	NO	NO	YES	YES	NO
Ints5	NO	NO	NO	YES	NO	NO	NO	NO	YES	NO
Ganab	NO	NO	YES	YES	NO	NO	NO	NO	YES	NO
B3gat3	NO	NO	YES	YES	NO	NO	NO	NO	YES	NO
Rom1	NO	YES	YES	YES	YES	NO	NO	YES	YES	YES
Eml3	NO	YES	YES	YES	NO	NO	NO	YES	YES	NO
Mta2	NO	YES	YES	YES	YES	NO	NO	YES	YES	YES
Tut1	NO	YES	YES	YES	YES	NO	NO	YES	YES	YES
Eef1g	NO	NO	YES	YES	NO	NO	NO	NO	YES	NO
Ahnak	NO	YES	YES	YES	YES	NO	NO	YES	YES	YES
Scgb1a1	NO	NO	YES	YES	YES	NO	NO	NO	YES	YES
Asrgl1	YES	YES	YES	YES	NO	NO	NO	YES	YES	NO
Gm6252	NO	NO	NO	NO	NO	NO	NO	NO	NO	NO
Stxbp3b	NO	NO	NO	NO	NO	NO	NO	NO	NO	NO
Incenp	NO	NO	YES	YES	NO	NO	YES	NO	YES	YES
Fth1	NO	YES	YES	YES	NO	NO	NO	YES	YES	NO
Best1	YES	YES	YES	YES	NO	NO	NO	YES	YES	NO
Rab3il1	YES	NO	YES	YES	NO	NO	NO	YES	YES	NO
Fads3	NO	YES	YES	YES	NO	NO	NO	YES	YES	NO
Fads2	YES	NO	YES	YES	NO	NO	NO	YES	YES	NO
Fads1	NO	YES	YES	YES	NO	NO	NO	YES	YES	NO
Fen1	NO	YES	YES	YES	NO	NO	NO	YES	YES	NO
Tmem258	NO	YES	NO	YES	NO	NO	NO	YES	YES	NO
Myrf	NO	NO	YES	YES	YES	NO	NO	NO	YES	YES
Dagla	YES	YES	YES	YES	NO	NO	NO	YES	YES	NO
Syt7	YES	NO	NO	YES	NO	NO	NO	YES	YES	NO
Lrrc10b	NO	YES	YES	YES	NO	NO	NO	YES	YES	NO
Ppp1r32	YES	YES	YES	NO	NO	NO	NO	YES	YES	NO
Sdhaf2	YES	NO	YES	YES	NO	NO	YES	YES	YES	YES
Cpsf7	NO	YES	YES	YES	YES	NO	NO	YES	YES	YES
Tmem216	NO	NO	YES	YES	NO	NO	NO	NO	YES	NO
Tmem138	NO	YES	NO	YES	NO	NO	NO	YES	YES	NO
Cyb561a3	NO	NO	NO	NO	NO	NO	NO	NO	NO	NO
Dak	YES	NO	NO	YES	NO	NO	NO	YES	YES	NO
Ddb1	YES	NO	YES	YES	NO	NO	YES	YES	YES	YES
Vwce	YES	NO	YES	YES	NO	NO	YES	YES	YES	YES
4930524O05Rik	NO	YES	NO	NO	NO	NO	NO	YES	NO	NO
Pga5	YES	NO	NO	NO	NO	NO	NO	YES	NO	NO
2210404E10Rik	NO	YES	NO	NO	NO	NO	NO	YES	NO	NO
Vps37c	NO	NO	YES	YES	NO	NO	NO	NO	YES	NO
Cd5	NO	NO	NO	YES	NO	YES	NO	NO	YES	YES
A430093F15Rik	NO	NO	NO	NO	NO	NO	NO	NO	NO	NO
Cd6	NO	YES	YES	YES	NO	NO	NO	YES	YES	NO
Slc15a3	YES	YES	YES	YES	NO	NO	NO	YES	YES	NO
Tmem132a	NO	NO	YES	YES	NO	YES	NO	NO	YES	YES
Tmem109	NO	NO	YES	YES	NO	YES	NO	NO	YES	YES
Prpf19	NO	NO	YES	YES	YES	NO	NO	NO	YES	YES
Zp1	NO	YES	NO	NO	NO	NO	NO	YES	NO	NO
Ptgdr2	NO	NO	NO	NO	NO	NO	NO	NO	NO	NO
Ccdc86	NO	YES	YES	YES	NO	NO	NO	YES	YES	NO
Ms4a10	YES	YES	NO	YES	NO	NO	NO	YES	YES	NO
Ms4a15	NO	YES	NO	NO	NO	NO	NO	YES	NO	NO
Ms4a18	NO	NO	YES	YES	NO	NO	NO	NO	YES	NO
Ms4a8a	NO	YES	NO	NO	NO	NO	NO	YES	NO	NO
1700017D01Rik	NO	YES	NO	NO	NO	NO	NO	YES	NO	NO
1700025F22Rik	NO	YES	NO	NO	NO	NO	NO	YES	NO	NO
Ms4a13	NO	YES	NO	YES	NO	NO	NO	YES	YES	NO
4930526L06Rik	NO	YES	NO	NO	NO	NO	NO	YES	NO	NO
Ms4a1	YES	YES	YES	YES	NO	NO	NO	YES	YES	NO
Ms4a5	YES	YES	NO	NO	NO	NO	NO	YES	NO	NO
Ms4a14	YES	NO	NO	NO	NO	NO	NO	YES	NO	NO
Ms4a7	NO	NO	NO	YES	NO	NO	NO	NO	YES	NO
Ms4a4c	NO	NO	NO	YES	NO	NO	NO	NO	YES	NO

Ms4a4b	NO	NO	YES	YES	NO	NO	NO	NO	YES	NO
Ms4a6c	NO	NO	YES	NO	NO	NO	NO	NO	YES	NO
Ms4a6b	YES	YES	NO	NO	NO	NO	NO	YES	NO	NO
Ms4a4d	YES	YES	NO	NO	NO	NO	NO	YES	NO	NO
Ms4a6d	YES	YES	NO	YES	NO	NO	NO	YES	YES	NO
Ms4a2	YES	NO	NO	NO	NO	NO	NO	YES	NO	NO
Ms4a3	YES	YES	NO	NO	NO	NO	YES	YES	NO	YES
Oosp2	NO	YES	NO	NO	NO	NO	NO	YES	NO	NO
Oosp1	NO	NO	NO	NO	NO	NO	NO	NO	NO	NO
Oosp3	YES	NO	NO	NO	NO	NO	NO	YES	NO	NO
Gif	NO	YES	YES	NO	NO	NO	NO	YES	YES	NO
Mrpl16	NO	YES	YES	YES	NO	NO	NO	YES	YES	NO
Stx3	YES	YES	YES	YES	NO	NO	YES	YES	YES	YES
Olfr1417	NO	YES	NO	NO	NO	NO	NO	YES	NO	NO
Olfr1418	YES	YES	NO	NO	NO	NO	NO	YES	NO	NO
Olfr1419	NO	YES	NO	NO	NO	NO	NO	YES	NO	NO
Olfr1420	NO	YES	NO	NO	NO	NO	NO	YES	NO	NO
Patl1	YES	YES	YES	YES	YES	NO	NO	YES	YES	YES
Osbp	YES	YES	YES	YES	NO	NO	NO	YES	YES	NO
Olfr1423	NO	NO	NO	NO	NO	NO	NO	NO	NO	NO
Olfr1424	NO	YES	NO	NO	NO	NO	NO	YES	NO	NO
Olfr1425	NO	YES	NO	YES	NO	NO	NO	YES	YES	NO
Olfr1426	NO	NO	NO	NO	NO	NO	NO	NO	NO	NO
Olfr1427	NO	NO	NO	NO	NO	NO	NO	NO	NO	NO
Olfr1428	NO	NO	NO	NO	NO	NO	NO	NO	NO	NO
Olfr76	NO	NO	NO	NO	NO	NO	NO	NO	NO	NO
Olfr1431	NO	YES	NO	NO	NO	NO	NO	YES	NO	NO
Olfr262	NO	NO	NO	YES	NO	NO	NO	NO	YES	NO
Olfr235	YES	YES	NO	YES	NO	NO	NO	YES	YES	NO
Olfr1433	YES	YES	NO	NO	NO	NO	NO	YES	NO	NO
Olfr1436	YES	NO	NO	NO	NO	NO	NO	YES	NO	NO
Olfr1437	NO	YES	NO	YES	NO	NO	NO	YES	YES	NO
Olfr1440	NO	NO	NO	NO	NO	NO	NO	NO	NO	NO
Olfr1441	YES	YES	NO	NO	NO	NO	NO	YES	NO	NO
Pfpl	YES	YES	YES	NO	NO	NO	NO	YES	YES	NO
Mpeg1	YES	YES	YES	NO	NO	NO	NO	YES	YES	NO
Dtx4	NO	YES	YES	YES	NO	NO	YES	YES	YES	YES
Fam111a	YES	YES	YES	NO	NO	NO	YES	YES	YES	YES
EG240549	NO	YES	NO	YES	NO	NO	NO	YES	YES	NO
Glyat	NO	NO	YES	NO	NO	NO	NO	NO	YES	NO
Olfr1442	NO	NO	NO	NO	NO	NO	NO	NO	NO	NO
Olfr1443	NO	NO	NO	NO	NO	NO	NO	NO	NO	NO
Keg1	NO	NO	YES	NO	NO	NO	NO	NO	YES	NO
Cntf	NO	NO	YES	YES	NO	YES	NO	NO	YES	YES
Zfp91-cntf	NO	NO	NO	NO	NO	NO	NO	NO	NO	NO
Zfp91	NO	YES	YES	YES	NO	NO	NO	YES	YES	NO
Lpxn	YES	YES	NO	NO	YES	NO	NO	YES	NO	YES
EG383436	NO	NO	NO	YES	NO	NO	NO	NO	YES	NO
Olfr1444	NO	NO	NO	YES	NO	NO	NO	NO	YES	NO
Olfr1445	NO	NO	NO	YES	NO	NO	NO	NO	YES	NO
Olfr1446	NO	NO	NO	YES	NO	NO	NO	NO	YES	NO
Olfr1447	NO	NO	NO	YES	NO	NO	NO	NO	YES	NO
Olfr1448	NO	NO	NO	NO	NO	NO	NO	NO	NO	NO
Olfr1449	NO	NO	NO	NO	NO	NO	NO	NO	NO	NO
Olfr1450	NO	NO	NO	NO	NO	NO	NO	NO	NO	NO
Olfr1451	NO	NO	NO	NO	NO	NO	NO	NO	NO	NO
Olfr1453	NO	YES	NO	YES	NO	NO	NO	YES	YES	NO
Olfr1454	NO	NO	NO	YES	NO	NO	NO	NO	YES	NO
Olfr1457	NO	NO	NO	NO	NO	NO	NO	NO	NO	NO
Olfr1459	NO	NO	NO	NO	NO	NO	NO	NO	NO	NO
Olfr1461	NO	NO	NO	NO	NO	NO	NO	NO	NO	NO
Olfr1462	NO	NO	NO	NO	NO	NO	NO	NO	NO	NO
Olfr1463	NO	NO	NO	NO	NO	NO	NO	NO	NO	NO
Olfr1465	NO	NO	NO	NO	NO	NO	NO	NO	NO	NO
Olfr1466	NO	NO	NO	NO	NO	NO	NO	NO	NO	NO
Olfr1467	NO	NO	NO	NO	NO	NO	NO	NO	NO	NO
Olfr1469	NO	NO	NO	NO	NO	NO	NO	NO	NO	NO
Olfr1471	NO	NO	NO	YES	NO	NO	NO	NO	YES	NO
Olfr1472	NO	NO	NO	NO	NO	NO	NO	NO	NO	NO
Olfr1474	NO	NO	NO	NO	NO	NO	NO	NO	NO	NO
Olfr1475	NO	NO	NO	NO	NO	NO	NO	NO	NO	NO
Olfr1477	NO	NO	NO	NO	NO	NO	NO	NO	NO	NO
Olfr1480	NO	NO	NO	NO	NO	NO	NO	NO	NO	NO
Olfr1484	NO	NO	NO	NO	NO	NO	NO	NO	NO	NO
Olfr1487	NO	NO	NO	NO	NO	NO	NO	NO	NO	NO
Olfr1489	NO	NO	NO	YES	NO	NO	NO	NO	YES	NO
Olfr1490	NO	NO	NO	NO	NO	NO	NO	NO	NO	NO
Olfr1491	NO	NO	NO	YES	NO	NO	NO	NO	YES	NO
Olfr1494	NO	NO	NO	YES	NO	NO	NO	NO	YES	NO
Olfr1495	NO	NO	NO	NO	NO	NO	NO	NO	NO	NO
Olfr1496	NO	NO	NO	NO	NO	NO	NO	NO	NO	NO
Olfr1497	NO	NO	NO	YES	NO	NO	NO	NO	YES	NO
Olfr1499	NO	NO	NO	NO	NO	NO	NO	NO	NO	NO
Olfr1500	NO	NO	NO	NO	NO	NO	NO	NO	NO	NO
Olfr1501	NO	NO	NO	NO	NO	NO	NO	NO	NO	NO
Olfr1502	NO	NO	NO	NO	NO	NO	NO	NO	NO	NO
Olfr1504	NO	NO	NO	NO	NO	NO	NO	NO	NO	NO
Olfr1505	NO	NO	NO	NO	NO	NO	NO	NO	NO	NO
Gm8630	NO	NO	NO	NO	NO	NO	NO	NO	NO	NO
Tle4	NO	NO	YES	YES	YES	NO	YES	NO	YES	YES
