# Supplementary material for: Quantitative trait loci on chromosomes 9 and 19 modulate AII amacrine cell number in the mouse retina
Source: Front Neurosci. 2023 Feb 2;17:1078168. doi: 10.3389/fnins.2023.1078168 (PMC9932814; doi:10.3389/fnins.2023.1078168)
Supplement: Supplementary file 3 [file Table_3.pdf]

Supplementary Table 3: Top Candidate Genes

Gene	Location (mm10)		Strand	Biological Process	Retinal Expression	High Priority Variants
**Ankk1**	Chr 9	49415194...49427041	(-)	Protein phosphorylation	Mature retina	Missense; 5'UTR; promoter (10)
**Cadm1**	Chr 9	47530170...47862280	(+)	Apoptotic process; cell adhesion; cell differentiation and growth; brain development; cell recognition	Developing AII amacrine cells; mature retina	Splice site (2); 3'UTR (4); promoter (3); structural variant (9)
**Cdon**	Chr 9	35421528...35507652	(+)	Cell fate; lens development; cell adhesion; neuron differentiation; cortex devlopment	Mature retina	Missense (2); splice site (3); 5'UTR; promoter
**Cryab**	Chr 9	50745951...50756636	(+)	Lens devlopment; gene expression; cell death; apoptosis	Mature retina	5'UTR; promoter (3)
**Dcps**	Chr 9	35124414...35175987	(-)	mRNA processing; RNA splcing; cell death regulation	Mature retina	5'UTR (4); promoter (8)
**Dixdc1**	Chr 9	50662752...50739602	(-)	Cell cycle; cortex development; cortex radially orientated cell migration; cell proliferation in forebrain; cytoskeleton organization; neuron differentiatoin; axonogensis; Wnt signalling	Developing retina; mature retina	Missense; splice site; 5'UTR (2); promoter (8)
**Drd2**	Chr 9	49340360..49408177	(+)	Synaptic transmission; GPCR signaling; dopaminergic; neuroblast proliferation	Developing retina; mature retina	Promoter (5)
**Fxyd2**	Chr 9	45399709..45410278	(+)	Cell growth; cell proliferation	Developing retina; mature retina	Promoter
**Kmt2a**	Chr 9	44803355..44881352	(-)	DNA methylation; transcription, cell proliferation; visual learning; response to light stimulus; post-embryonic development; synaptic plasticity; histone methylation/acylation	Developing retina; mature retina	Promoter
**Ncam1**	Chr 9	49502129..49799393	(-)	Cell adhesion; axon guidance; neuron projection development; neuron development; cell death regulation; synaptic plasticity	Developing AII amacrine cells; mature retina	3'UTR; promoter (3); structural variant (2)
**Pou2af1**	Chr 9	51213690..51240079	(+)	Positive regulation of transcription	Developing retina	Promoter (3)
**Ppp2r1b**	Chr 9	50856896..50899325	(+)	Apoptotic process in morphogenesis	Developing retina; mature retina	3'UTR (4); promoter (10)
**Rbm7**	Chr 9	48488697..48495330	(-)	Nucleotide binding; nucleic acid binding; RNA binding	Developing AII amacrine cells; mature retina	Promoter
**Rdx**	Chr 9	52047150..52088738	(+)	Regulation of cell shape and size; regulation of gene expression; actin bundle assembly; cell migration; cell adhesion; mitosis	Developing AII amacrine cells; mature retina	Splice site; 3'UTR (3); promoter (5)
**Tirap**	Chr 9	35184391..35200291	(-)	Signal transduction; immune/inflammation	Developing retina; mature retina	3'UTR (3); promoter
**Trim29**	Chr 9	43310715..43336125	(+)	Negative regulation of transcription	Mature retina	3'UTR (3); splice site
**Usp28**	Chr 9	48985319..49042517	(+)	DNA repair; cell proliferation	Developing retina; mature retina	Promoter (3)
**Zbtb16**	Chr 9	48651797..48836225	(-)	Transcription regulation; apoptotic regulation; CNS development; cell proliferation	Developing retina; mature retina	5'UTR (3); structural variant (2)
**Zw10**	Chr 9	49055576..49078775	(+)	Mitosis; cell cycle	Developing retina; mature retina	Missense; splice site; promoter; structural variant
**Ahnak**	Chr 19	8989284..9076935	(+)	RNA splice regulation	Developing retina; mature retina	Promoter
**Cpsf7**	Chr 19	10525223..10547735	(+)	mRNA processing	Developing AII amacrine cells; mature retina	Promoter
**Ddb1**	Chr 19	10605568..10629828	(+)	DNA repair; Wnt signaling; mitotic phase transition	Developing AII amacrine cells; mature retina	Splice site
**Dtx4**	Chr 19	12466329..12502179	(-)	Notch signaling	Developing retina; mature retina	3'UTR; structural variant
**Fam111a**	Chr 19	12573510..12589769	(+)	DNA replication	Developing retina; mature retina	Missense (46); 3'UTR (2), promoter, structural variant
**Mta2**	Chr 19	8941920..8952300	(+)	Transcription regulation; DNA methylation and packaging	Developing retina; mature retina	Promoter
**Patl1**	Chr 19	11912399..11945096	(+)	Translation regulation; mRNA processing and regulation	Developing retina; mature retina	Missense; splice site; 3'UTR , promoter; structural variant
**Rom1**	Chr 19	8927382..8929356	(-)	Cell adhesion; visual percention; gene expression regulation; photoreceptor differentiation; retinal vasculature development	Developing retina; mature AII amacrine cells	Promoter
**Sdhaf2**	Chr 19	10500512..10525209	(-)	Wnt signaling	Developing retina; mature retina	Missense

**Stx3**	Chr 19	11775118..11821569	(-)	Cell proliferation	Developing AII amacrine cells; mature retina	Missense; splice site (2); 3'UTR (12); 5'UTR (5); promoter (16)
**Tut1**	Chr 19	8953832..8966210	(+)	mRNA and snRNA processing	Developing retina; mature retina	Promoter (2)
**Vwce**	Chr 19	10634218..10666213	(+)	Cell proliferation; signal transduction	Developing retina	Missense (2); splice site
**Zbtb3**	Chr 19	8802489..8804853	(+)	Transcription	Mature retina	Missense; promoter
