# Supplementary figures and images for: Quantitative trait loci on chromosomes 9 and 19 modulate AII amacrine cell number in the mouse retina
Source: Front Neurosci. 2023 Feb 2;17:1078168. doi: 10.3389/fnins.2023.1078168 (PMC9932814; doi:10.3389/fnins.2023.1078168)

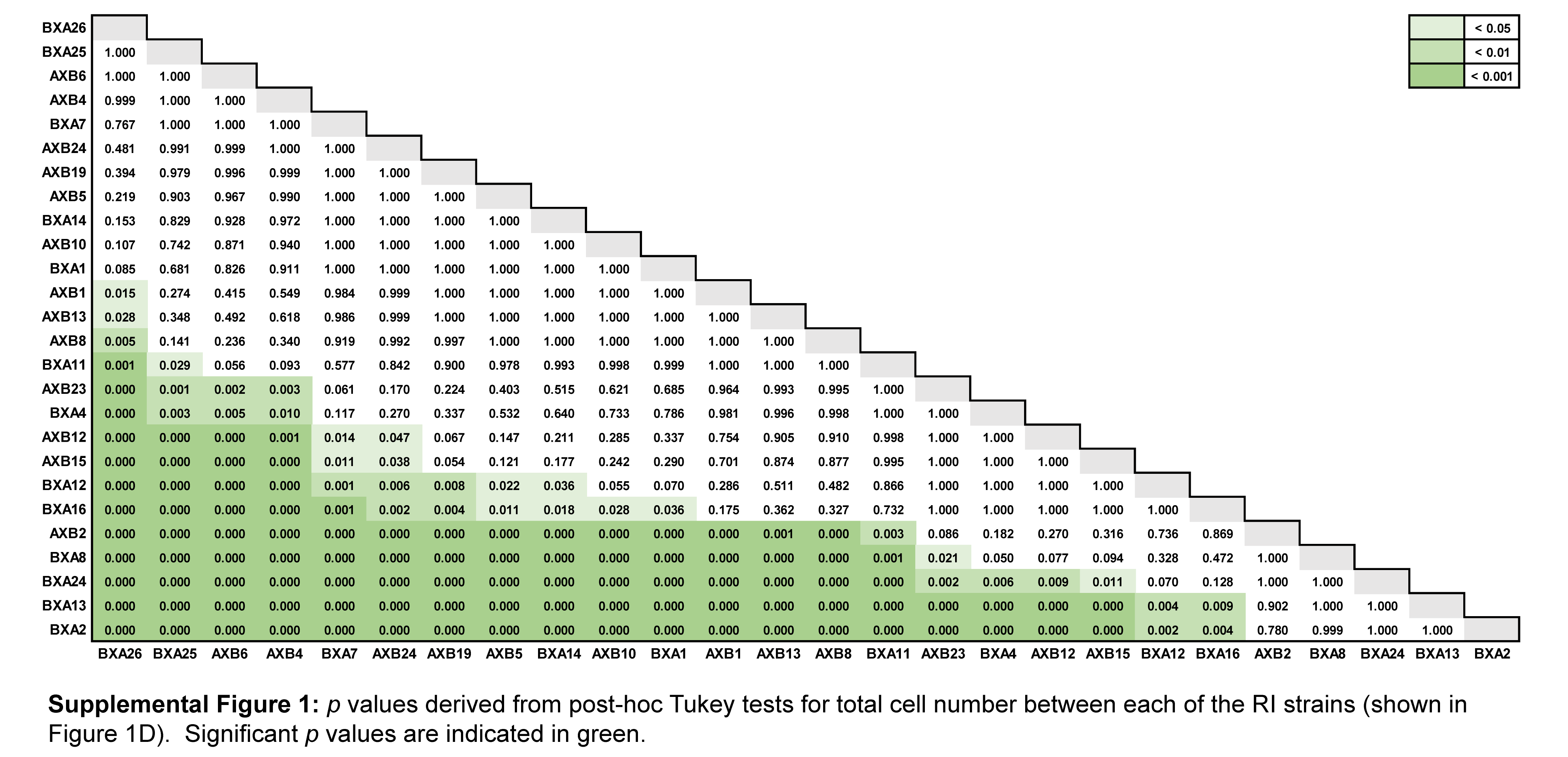

Supplement: Supplementary file 4 [file Image_1.TIF]
